# Daily affect intensity and variability of adolescents and their parents before and during a COVID‐19 lockdown

**DOI:** 10.1002/jad.12117

**Published:** 2022-11-07

**Authors:** Lianne P. de Vries, Anne Bülow, Dirk H. M. Pelt, Savannah Boele, Meike Bartels, Loes Keijsers

**Affiliations:** ^1^ Department of Biological Psychology Vrije Universiteit Amsterdam Amsterdam The Netherlands; ^2^ Amsterdam Public Health Research Institute Amsterdam University Medical Centres Amsterdam The Netherlands; ^3^ Department of Psychology, Education and Child Studies Erasmus University Rotterdam Rotterdam The Netherlands

**Keywords:** affect, COVID‐19, daily diary, lockdown, piecewise growth models, well‐being

## Abstract

**Introduction:**

The corona virus (COVID‐19) pandemic may have a prolonged impact on people's lives, with multiple waves of infections and lockdowns, but how a lockdown may alter emotional functioning is still hardly understood.

**Methods:**

In this 100‐daily diaries study, we examined how to affect intensity and variability of adolescents (*N* = 159, *M*_age_ = 13.3, 61.6% female) and parents (*N* = 159, *M*_age_ = 45.3, 79.9% female) changed after the onset and during (>50 days) the second COVID‐19 lockdown in the Netherlands, using preregistered piecewise growth models.

**Results:**

We found only an unexpected increase in parents' positive affect intensity after the lockdown onset, but no immediate changes in negative affect intensity or variability. However, both adolescents and parents reported gradual increases in negative affect intensity and variability as the lockdown prolonged. Lockdown effects did not differ between adolescents and parents. However, within groups, individuals differed. The individual differences in the effects were partly explained by life satisfaction, depressive symptoms, and self‐reported lockdown impact.

**Conclusions:**

Overall, these findings suggests that a lockdown triggers changes in daily affective well‐being especially as the lockdown prolongs. Individual differences in the effects indicate heterogeneity in the impact of the lockdown on daily affect that was partly explained by baseline life satisfaction and depressive symptoms. However, more knowledge on the causes of this heterogeneity is needed to be able to increase resilience to lockdown effects in the population.

## 1 INTRODUCTION

The global corona virus (COVID‐19) pandemic has a large and prolonged impact on people's everyday lives. During the first year of the pandemic, there was no effective cure, and prolonged restrictions were needed to control the virus, and make sure the healthcare systems could cope. Major restrictions during lockdowns included social distancing, and the closing of schools, offices, and public places. The restrictions in combination with distress about the virus led, on average, to small increases in anxiety and depressive symptoms and decreases in well‐being and life satisfaction (Masten & Motti‐Stefanidi, 2020; Prati & Mancini, 2021; Robinson et al., 2022; Weeland et al., 2021). However, the negative effect of this first phase of the pandemic disappeared quickly: by mid‐2020 average mental health had recovered to prepandemic levels (Robinson et al., 2022). These findings indicate that, in line with earlier findings in the field of resilience (Galatzer‐Levy et al., 2018), most people were relatively resilient to the effects of the initial phase of the pandemic with respect to their well‐being. Most people appear to be able to adapt quickly to the new situation. However, it has also been demonstrated that individuals differ in the extent to which the lockdown affects their daily life, relationships, and well‐being (Bülow et al., 2021; de Vries et al., 2021; Janssen et al., 2020; Santomauro et al., 2021; van de Weijer et al., 2022). Younger age groups, such as adolescents, may be more vulnerable, as they still have to build adaptive capacity to cope with such restrictions (Masten, 2021; Masten & Motti‐Stefanidi, 2020; Santomauro et al., 2021).

Whereas research on the first lockdown led to knowledge about the acute response to the pandemic and feelings of uncertainty that people experienced about the virus, many countries experienced multiple waves of COVID‐19 infections resulting in multiple lockdowns. Compared to the first lockdown, uncertainty about the virus might have decreased. In Germany, Austria, and Australia, for instance, individuals reported on average increased depressive symptoms and higher psychological burden during the second, less strict, lockdown compared to the first lockdown (Büssing et al., 2021; Dale et al., 2021; Johnston & Oliva, 2021; Moradian et al., 2021). Especially the 18–24 year olds showed a large increase in depressive symptoms compared to the first lockdown (Dale et al., 2021). Explanations for these findings could include a general mental exhaustion or “pandemic fatigue” as the pandemic and societal disruption continued (WHO, 2020). Furthermore, negative economic effects increased as well during the pandemic, leading to potential higher psychological burden for affected individuals. Investigating the effect of later lockdowns on well‐being helps to understand the more prolonged effects, and could inform policy with regard to the expected psychological impact of future lockdowns. In the current preregistered 100‐day diaries study, we therefore examined (1) the effects of a second lockdown in the Netherlands (December 2020–March 2021) on everyday positive and negative affect intensity and variability, (2) how these effects differed between adolescents and adults, that is, their parents, (3) which individuals were most vulnerable to the impact of a lockdown by investigation of individual differences, and (4) how these differences related to baseline life satisfaction, depressive symptoms and self‐reported impact of the lockdown on daily life.

### 1.1 Changes in daily positive and negative affect intensity and variability

Although evidence for the pandemic's impact upon general mental health and well‐being is accumulating, less is known about the more subtle or dynamic effects on daily affective well‐being. Affective well‐being can be defined by the frequent experience of high positive affect and low negative affect (Diener et al., 2018). Using the experience sampling method (ESM) in which affective well‐being is assessed multiple times per day, or every day with a daily diary design, will give insights in the *daily affect intensity* and *affect variability*. Daily affect intensity can be defined as the level of daily positive or negative affect. However, feelings of positive and negative affect are not stable and fluctuate over time (i.e., across the day and week) and across different contexts (Eid & Diener, 2004; Li et al., 2014). Therefore, it is equally important to understand how everyday well‐being fluctuates from one moment to the next, which we call affect variability. Individuals differ in their affect variability, some individuals show relatively stable positive and negative affect levels over the day or week, while others fluctuate considerably (Eid & Diener, 1999; Gadermann & Zumbo, 2007; Kuppens et al., 2010). Within boundaries, affect variability is adaptive and important for well‐being as it helps to respond to environmental changes and demands (Carver, 2015; Frijda & Mesquita, 1994; Kashdan & Rottenberg, 2010). However, if emotions change too strongly or not at all it may signal dysregulation. Large variability of positive and negative affect have been associated with reduced well‐being and increased risk for mental health problems (Aan het Rot et al., 2012; Houben et al., 2015; Maciejewski et al., 2019; Reitsema et al., 2022; Schoevers et al., 2021). Thus, in the context of this study, changes in affect dynamics during the COVID‐19 pandemic could be an early marker for increased risk to develop emotional problems.

Few studies have investigated how the pandemic and lockdown has altered daily affect intensity and variability. Two studies reported a decrease in daily positive affect intensity and an increase in negative affect intensity during the pandemic and lockdown (Deng et al., 2021; Green et al., 2021). Regarding affect variability, mixed findings were reported, that is, a decrease in positive affect variability, but not negative affect variability (Deng et al., 2021; Green et al., 2021), or no change in affect variability at all (Asscheman et al., 2021). The first aim of the current study was to examine the effects of a lockdown on daily positive and negative affect intensity and affect variability. Based on previous studies, we expected that daily negative affect would increase, and positive affect intensity decrease (H1a). Moreover, we expected a decrease in positive affect variability at the start of the lockdown (compared to before) (H1b). We did not have a clear hypothesis about the negative affect variability because of the inconsistencies in the literature. Moreover, we explored the gradual changes in daily affect intensity and variability during the first 7 weeks of the lockdown. To test these hypotheses, the current study examines data of 100 consecutive days, in which adolescents and one of their parents reported on their daily positive and negative affect. The first 50 days were before the second lockdown in the Netherlands and the last 50 days were during the second lockdown.

### 1.2 Difference between adolescents and parents

The lockdown affects the whole family system, including adolescents and their parents (Bülow et al., 2021; Masten, 2021; Weeland et al., 2021). Yet, compared to their parents, adolescents could be more strongly affected by the lockdown (Fegert et al., 2020), because of several reasons. First, adolescence can by itself be a stressful period, which is characterized by substantial life changes, and the development of identity and emotion regulation (Maciejewski et al., 2019; Zeman et al., 2006). Second, during lockdowns, adolescents experience many restrictions which can possibly affect their development, such as school closure, and restrictions in social and sports activities. Moreover, in the Netherlands, different restrictions were established for different age groups during the time between the first (March–June 2020) and the second lockdown (December 2020–March 2021). In these months *before* the second lockdown, adolescents were less restricted than adults. For adolescents (<18 years), schools and sports clubs were kept open and adolescents did not have to keep distance from each other, whereas adults had to keep 1.5 m distance and could not meet to sport together. However, *during* the second lockdown, the rules for adolescents and adults were equally strict, that is, both groups had to keep 1.5 m distance and public spaces, schools, and shops were closed. Therefore, for adolescents, the transition from the relatively measure‐free period before the lockdown to the strict lockdown period might be larger compared to the transition for adults. This difference could make the impact of a second lockdown potentially larger for adolescents than for their parents (see Table 1 for an overview of the restrictions before and during the second lockdown separately for adolescents and adults).

**Table 1:** Important COVID‐19‐related restrictions during the study period, before (October 26–December 14, 2020) and during the second lockdown (December 15, 2020–March 1, 2021) in the Netherlands, separately for adolescents and adults

	Adolescents (<18)	Adults (≥18)	General
Before 2nd lockdown (Oct 26, 2020–Dec 14, 2020)	No 1.5 m distance	1.5 m distance	Public places open
	Schools open	Working from home	Shops open
	Sport clubs open	Sport with maximum of 4 people with 1.5 m distance	
During 2nd lockdown (Dec 15, 2020–March 1, 2021)	1.5 m distance	1.5 m distance	Public places closed
	Homeschooling	Working from home	Nonessential shops closed
	Sportsclubs closed	Sportclubs closed	Jan 23: curfew

A recent study suggests that mainly adolescents are at risk for emotional problems due to COVID‐19 and lockdowns (Santomauro et al., 2021). However, differences between adolescents and adults have not been examined by direct comparisons. Therefore, our second aim was to compare the lockdown effect on positive and negative affect intensity and variability between adolescents and their parents. Specifically, we expected adolescents to be more strongly affected by the lockdown than their parents, as reflected in stronger effects on affect intensity and variability, because of differences in developmental tasks and restrictions (H2).

### 1.3 Individual differences

All psychological processes are heterogeneous (Bolger et al., 2019) and individuals also vary in their responses to stressful life events, like the COVID‐19 pandemic (Galatzer‐Levy et al., 2018; Mancini, 2020). Most people show resilience and adapt relatively quickly to new situations, whereas less resilient people do not cope well in response to stress and experience long‐term adverse effects, that is, lower well‐being and possible development of psychopathology (Bonanno et al., 2011; Galatzer‐Levy et al., 2018). Indeed, empirical studies demonstrate such individual differences in the effects a lockdown had on daily life and well‐being (de Vries et al., 2021; Janssen et al., 2020; Santomauro et al., 2021; van de Weijer et al., 2022). Therefore, our third aim was to investigate how the impact of lockdown on daily affect intensity and variability would differ between individuals (i.e., effect heterogeneity; Bolger et al., 2019). Individual differences were hypothesized for all effects (H3). For example, we expected some participants to show an increase in negative affect intensity as a result of the lockdown, whereas others will show a decrease.

Furthermore, we explored why some individuals were more strongly affected, by assessing relevant moderators. Because well‐being and mental health have been related to resilience and to positive and negative affect intensity and variability (Houben et al., 2015; Reitsema et al., 2022), we assessed baseline (i.e., before the second lockdown, October 2020) life satisfaction and depressive symptoms. We expected that individuals with a higher baseline life satisfaction and fewer depressive symptoms would report overall less positive and negative affect variability (H4a). Moreover, we expected that lower life satisfaction and a higher level of depressive symptoms at baseline would be related to larger changes in affect intensity and variability, that is, indicating a larger impact of the lockdown (H4b). Finally, we explored the relation between the self‐reported lockdown impact on daily life and affect intensity and variability.

## 2 METHODS

This study was preregistered using a template for experience sampling methodology (ESM) research (Kirtley et al., 2021) (https://osf.io/8s6vn).

### 2.1 Participants

We used existing data of the “100 days of my life” study, including daily data of 159 adolescents and one of their parents (Bülow et al., 2020, https://osf.io/5mhgk/). Inclusion criteria were, that (1) adolescents were aged 12–16 years, (2) parents and adolescents owned a smartphone, and (3) parents and adolescents had contact with each other almost every day (e.g., living together). Adolescents were on average 13.3 years old (SD = 1.2, range = 12–16 years) and 61.6% was female (36.5% male, 1.9% other). Most adolescents were born in the Netherlands (88.9%) and followed preuniversity high school (VWO) (50.9%), whereas 28.9% of the adolescents was in higher general high school (HAVO), 15.1% followed prevocational high school (VMBO) and 5.0% was in a mixed track (VMBO/HAVO or HAVO/VWO). Parents were on average 45.3 years old (SD = 4.5, range = 33–55 years) and mostly female (79.9%). Most parents were born in the Netherlands (86.8%) and were highly educated with either a college or university degree (62.7%), whereas 25.3% of the parents had a vocational/technical training and 10.1% was low‐educated (i.e., high school diploma).

### 2.2 Procedure

Participants were recruited at two Dutch high schools (via e‐mails, social media, posters, and class visits) and via newsletters. During 100 consecutive days (October 26, 2020, until February 2, 2021), both adolescents and their parents received daily questionnaires via the Ethica application on their Android or iOS smartphone (Ethica Data, 2020). Participants could choose when they wanted to receive the daily notification (between 7 p.m. and 10 p.m.). Participants were reminded three times (every 30 min) and one time the next morning (7 a.m.) to answer the questionnaire. Participants could answer up to 12 p.m. the next day. In every daily questionnaire, participants answered 20–27 items, which took approximately 3–5 min. The compliance rate was high, with 89% and 94% completed by respectively the adolescents and parents.

Additionally to the daily questionnaires, participants answered five longer questionnaires in Qualtrics (2021) every 3 months, with the first at the start of the 100 days period. In this study, we included life satisfaction and depressive symptoms from the baseline questionnaire (October 2020) and self‐reported lockdown impact from the second longitudinal questionnaire (January 2021).

Participants received a monetary reward for answering each questionnaire and gained bonuses if they answered 10 questionnaires in a row or answered all 100‐daily questionnaires. In total, adolescents could earn 100€ (approx. US$ 116) and parents 50€ (approx. US$ 58). Every day, adolescents could win 10€ (approx. US$ 12) if they answered the questionnaire. Adolescents could choose to get a diary booklet with their own answers at the end of the study. The study and amendments of the design due to the lockdown were approved by the ethical committee of Tilburg University (RP250).

**Figure 1:**
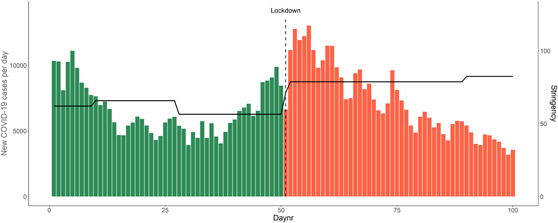
Timeline of the number of COVID‐19 cases per day (left *y*‐axis) and the stringency of the measures (right *y*‐axis) in the Netherlands during the 100‐day study period. The stringency index is a measure of the strictness of the COVID‐19 regulations, based on school closure, workplace closure, and travel bans (ranging from 0 to 100). *Source*: https://ourworldindata.org/grapher/covid-stringency-index?region=Europe&country=%7ENLD [Color figure can be viewed at wileyonlinelibrary.com]

Halfway during the data collection, at Day 51 (December 15, 2020), the government introduced the second strict lockdown in the Netherlands, which lasted up to Day 100 of the study. As shown in Figure 1, on the first day of the data collection (October 26, 2020) the restrictions were less strict, schools and sports clubs were open and adolescents until 18 years did not have to distance themselves from each other. Adults (≥18 years) were required to keep 1.5 m distance from everyone, besides their own household, and were required to work from home. During the strict lockdown, schools, public places, and nonessential shops were closed (see Figure 1 and Table 1). After the study period, the restrictions of the lockdown were released, that is, on March 1st high schools and shops opened again.

### 2.3 Measures

#### 2.3.1 Daily positive and negative affect

Daily positive and negative affect were assessed with a shortened version of the Positive and Negative Affect Scale for Children (PANAS‐C) (Ebesutani et al., 2012). Items included were joyful (Dutch: *blij*) and happy (*gelukkig*) for positive affect and mad (*boos*), afraid (*angstig*), and sad (*verdrietig*) for negative affect. Participants had to rate on a visual analogue scale from 0 (*not at all*)–100 (*very much*) to what extent they felt these emotions during the whole day. The selected items were chosen based on previous work (Bülow et al., 2022; Vogelsmeier et al., 2021). The within‐person correlation for the two positive affect items was high for both adolescents (*r* = .75) and their parents (*r* = .83). The within‐person and between‐person reliability of the three negative affect items was good for both adolescents (*ω*_within_ = 0.71, *ω*_between_ = 0.92) and parents (*ω*_within_ = 0.68, *ω*_between_ = 0.80) (Geldhof et al., 2014).

#### 2.3.2 Affect intensity and variability

To investigate how positive and negative affect intensity and variability changed, we computed average intensity and variability per week for every participant (*n*_total weeks_ = 2192 and 2206 for adolescents and parents, respectively). Hence, 14 data points per variable were obtained, that is, 7 weeks before the lockdown and 7 weeks during the lockdown. Affect intensity was computed as the average score across 7 days separately for positive and negative affect. Higher scores indicate higher positive or higher negative affect. We defined the variability of positive and negative affect as the square root of the mean square of successive differences (rMSSD), computed as

$$\mathrm{rMSSD}=\sqrt{\frac{\sum_{i=1}^{n-1}{\left(x_{i+1}-x_{i}\right)}^{2}}{n-1},}$$

using the R function rMSSD (Jahng et al., 2008; Von Neumann et al., 1941). The rMSSD is often referred to as affective instability (Koval et al., 2013; Trull et al., 2015). Larger rMSSD reflects higher day‐to‐day variability, whereas smaller rMSSD indicates less variability (see Supporting Information: Figure S1, for examples of high and low affect intensity and variability). Following Koval et al. (2013), we first computed the rMSSD of each positive and negative affect item and then averaged the rMSSD for all the positive items as the final score for positive affect variability and the negative items as the score for negative affect variability. The rMSSD is strongly related to the SD (Dejonckheere et al., 2019; Wendt et al., 2020), which was also the case in the current study (*r* > .85). We used the rMSSD instead of SD to measure variability, because SD reflects only the size of variability, whereas the rMSSD also takes into account temporal dependence between subsequent moments (Jahng et al., 2008).

#### 2.3.3 Baseline measures

##### Adolescents' life satisfaction

For adolescents, the Cantril's Self‐Anchoring Ladder (Levin & Currie, 2014) was used to measure life satisfaction. Using a single‐item measure, participants had to indicate their quality of life on a scale from 1 to 10, with a score of 10 indicating the best quality.

##### Adolescents' depressive symptoms

Adolescents reported their depressive symptoms by using the Reynolds Adolescent Depression Scale Short (RADS‐2) (Reynolds, 2004). Participants had to the rate 10 items about occurrence of certain behavior or feeling on a scale from 1 (*almost never*) to 4 (*often*). An example item is *I've felt like nothing I was doing made sense*. The Cronbach's *α* in the current study was 0.90 (95% confidence interval [CI]: 0.87–0.92), indicating excellent internal consistency.

##### Parents' depressive symptoms

Parents reported the presence of depressive symptoms by using the Brief Depression Inventory (BDI) (Beck & Beck, 1972). The BDI included 21 items about how the parent has been feeling in the last week. For every question, parents had to choose among four statements (0–3) and pick the one that resembled their feelings the most. For example, an item is *I do not feel like a failure* (0) up to *I feel I am a complete failure as a person* (3). The Cronbach's *α* in the current study was 0.84 (95% CI: 0.80–0.87), indicating good internal consistency.

##### Self‐reported impact of the lockdown

During the second longitudinal questionnaire (end of January), adolescents and their parents completed the COVID‐19 Impact Questionnaire. This questionnaire was based on items from other surveys (Achterberg et al., 2021; Brown et al., 2020; Conway et al., 2020; Ellis et al., 2020; Magson et al., 2021) and included seven items on the impact of the lockdown on their daily life, well‐being, financial situation, school performance, and relations with family and friends. Participants answered on a scale from −3 (*very negative*) to +3 (*very positive*). We summed the scores on these seven items to create a score for self‐reported impact. Larger negative scores indicate a larger negative effect on daily life and well‐being. The Cronbach's *α* of this scale was 0.78 (95% CI: 0.73–0.84) for adolescents and 0.74 (95% CI: 0.68–0.80) for parents, indicating good internal consistency.

##### Coping

During the second longitudinal questionnaire (end of January), adolescents and their parents also completed the short version Utrecht coping list (UCL) (Schreurs et al., 1993). The UCL consist of 19 items that measure four different coping strategies in response to problems or difficult events, namely confrontation, avoidance, social support, and palliative reaction. Participants answered on a scale from 1 (*seldom*) to 4 (*very often*) how often they applied the strategy when confronted with problems. We summed the scores on the items per strategy to create a score for the four different coping strategies. Larger scores indicate more use of the particular coping strategy.

### 2.4 Statistical analyses

#### 2.4.1 Piecewise growth models

To compare the change in positive and negative affect intensity and variability from prelockdown to the lockdown period, we applied latent piecewise growth models (Bülow et al., 2021; Flora, 2008) following our preregistered plan (https://osf.io/8s6vn). Piece‐wise models allow to disentangle more sudden environmental changes from the ongoing trajectory of change by adding additional growth factors. Conceptually, modeling the within‐person change (while controlling for stable between‐person differences), each individual has its own “control condition”: the estimated prelockdown level of affect and change trajectory if the lockdown had not occurred. We ran eight models, namely models with positive and negative affect intensity and variability as outcomes (2 × 2) separately for adolescents and parents (×2). Several sensitivity analyses replicated our main results (for more detail see Supporting Information Materials).

**Figure 2:**
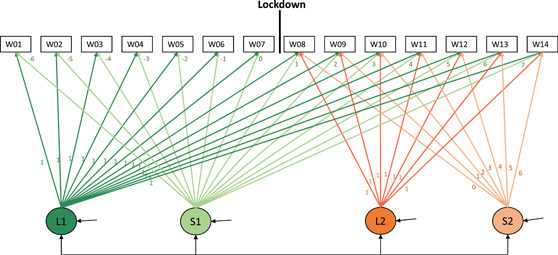
Piecewise growth model (Flora, 2008), based on Bülow et al. (2021) with an intercept and slope to model change during the whole study period (L1 and S1) and an intercept and slope to capture the additional impact of the lockdown period (L2 and S2), starting at week 8. W01–W14 indicates the positive or negative affect intensity or variability score of Week 1 until Week 14 (based on 100 days of data). [Color figure can be viewed at wileyonlinelibrary.com]

In the models, we modeled ongoing levels and changes by estimating a baseline intercept (level 1: L1) and slope (S1) for the whole study period. The factor loadings of S1 were centered at the week before the lockdown, and, therefore, the intercept (L1) can be interpreted as the affect intensity or variability directly before the lockdown. The slope S1 reflects linear changes in affect intensity or variability during the whole study period (Week 1–14). To assess how the lockdown period altered this trajectory, we added a second intercept (level 2: L2) and slope (S2). The loadings of S2 are centered at Week 8, that is, the first lockdown week. Therefore, the second intercept (L2) reflects the level difference between affect intensity or variability the week before the lockdown and the first lockdown week. S2 reflects the linear changes specifically during the lockdown (Week 8–14), over and above the normative trajectory as modeled by S1. We allowed the intercepts and slopes to vary across individuals by adding their variances and added the correlations between random intercepts and slopes (see Figure 2 for the model). All available data were used. To deal with missing data, the maximum likelihood for robust standard errors (MLR) estimation (MLR) was used. In sensitivity models, we tested the effect of stricter thresholds for data inclusion, and the results did not differ (for more detail see Supporting Information Materials). The model fit for the models was evaluated based on the root mean square error of approximation (RMSEA) (<0.08), comparative fit index (CFI) (>0.90) and Tucker–Lewis index (TLI) (>0.90) (Hu & Bentler, 1999; McDonald & Ho, 2002). We interpreted the model findings if two of the three fit measures were acceptable. If not specified otherwise, in all tests, we used *p* < .05 as inference criteria.

Aim 1. To test the effect of the lockdown on the daily affect intensity or variability (H1), we examined the significance of the second intercept (L2). Moreover, we explored the significance of the slope during lockdown (S2).
Aim 2. To assess differences between adolescents and their parents in the lockdown effect on affect variability and intensity (H2), we constrained the intercept and slope for the lockdown (L2 and S2) of the parents to be equal to the values of the adolescents' model and compared the model fit using *χ* ^2^ difference tests.
Aim 3. To investigate individual differences within the groups in changes in the affect intensity or variability (H3), we assessed whether the variance around the second intercept (L2) and the second slope (S2) is significant (one‐tailed *p* < .05).
Aim 4. To understand why the lockdown may impact individuals differentially, we further added life satisfaction (only for adolescents) and depressive symptoms (for adolescents and parents) as predictors to additional piecewise growth models. To test the overall association between affect intensity or variability and life satisfaction or depressive symptoms, we tested the significance of the association between the predictor and the intercept L1 (H4a). To investigate the association with the lockdown effect on affect intensity and variability, we tested the significance of the association between the predictor and lockdown intercept L2 and slope S2 (H4b).

Similarly, as an exploratory (i.e., not preregistered) analysis, we included self‐reported impact of the lockdown and the four coping strategies as predictor in the models. We tested the significance of the association between the lockdown impact or coping strategies and the intercepts and slopes.

## 3 RESULTS

In Table 2, the descriptive statistics and average positive and negative affect intensity and variability during the weeks before and during the lockdown can be found. The variables were normally distributed (skewness < 3.00 and kurtosis < 10.00).

**Table 2:** Descriptives of positive and negative affect intensity and variability

Week	*n*	PA intensity	SD PA intensity	NA intensity	SD NA intensity	PA variability	SD PA variability	NA variability	SD NA variability
Adolescents									
1	159	75.02	16.18	12.66	16.18	15.67	10.36	14.76	10.36
2	159	77.49	16.65	11.67	16.65	12.96	8.57	12.69	8.57
3	159	76.68	17.71	11.70	17.71	11.77	8.80	12.12	8.80
4	158	75.46	18.76	11.51	18.76	12.28	9.81	12.08	9.81
5	159	75.38	18.64	11.14	18.64	12.25	10.78	11.17	10.78
6	158	76.93	17.79	10.35	17.79	11.24	9.34	10.38	9.34
7	157	75.70	18.32	11.61	18.32	10.78	9.82	11.16	9.82
Lockdown									
8	156	76.79	18.21	10.47	18.21	10.82	9.48	9.66	9.48
9	157	78.62	18.17	10.43	18.17	9.45	8.92	8.81	8.92
10	154	78.67	17.87	9.34	17.87	9.17	9.59	7.87	9.59
11	154	75.15	17.81	11.28	17.81	10.90	9.50	10.25	9.50
12	155	75.58	18.80	11.59	18.80	9.46	9.10	9.65	9.10
13	155	76.05	19.23	10.57	19.23	8.91	9.16	9.00	9.16
14	152	76.57	17.73	11.58	17.73	8.57	8.80	8.67	8.80
Parents									
1	159	70.3	70.27	15.18	11.63	9.29	14.90	8.20	12.98
2	159	70.3	70.28	16.28	11.45	11.01	13.02	7.89	11.32
3	159	71.0	71.05	15.11	9.94	8.94	12.24	8.25	10.73
4	159	69.6	69.57	16.45	10.40	10.07	11.79	8.20	10.60
5	159	70.1	70.13	16.94	9.45	9.42	11.24	8.39	9.53
6	159	70.8	70.81	17.39	9.65	10.95	11.21	9.54	9.38
7	158	69.7	69.70	17.66	9.80	10.49	10.15	7.80	9.13
Lockdown									
8	158	68.8	68.84	17.34	11.35	10.63	10.50	8.08	10.73
9	156	72.3	72.27	17.12	8.65	9.00	10.05	7.76	8.50
10	157	72.0	72.00	17.17	8.97	9.77	9.96	8.28	8.23
11	156	70.3	70.29	17.30	9.93	10.65	9.89	6.74	8.03
12	155	69.0	69.02	17.78	10.52	10.75	9.27	6.97	8.73
13	156	68.9	68.90	17.51	10.59	10.22	9.97	8.48	9.54
14	156	68.9	68.86	18.11	11.60	11.75	9.81	7.48	10.02

Abbreviations: PA, positive affect, NA, negative affect.

### 3.1 Aim 1: Changes in daily affect intensity and variability

All preregistered piecewise growth models had an acceptable model fit with maximum likelihood for robust standard errors (MLR) estimation (RMSEA =  0.04–0.10, CFI > 0.90, and TLI > 0.90, Supporting Information: Table S1). Covariance matrices of all models can be found in Supporting Information: Tables S11–S18.

The first hypothesis (H1a) of direct decreases and increases in respectively daily positive affect and negative affect during the second COVID‐19 lockdown was not supported. Instead, we found a small increase in positive affect intensity for parents (*M*_L2_ = 1.75, SE = 0.63, *p* = .005) (see Table 3, L2). We also did not find the expected decrease in positive affect variability due the lockdown (H1b) (see Table 3, L2).

**Table 3:** Piecewise growth models to assess the impact of a lockdown on adolescents and parents' positive and negative affective well‐being

		Adolescents						Parents					
Variable		Mean	SE	*p*	Variance	SE	*p*	Mean	SE	*p*	Variance	SE	*p*
PA intensity	L1	76.26	1.44	<.001	312.04	37.06	**<.001**	70.06	1.39	<.001	290.35	34.30	**<.001**
	S1	0.03	0.14	.819	1.60	0.36	**<.001**	−0.08	0.15	.599	2.05	0.40	**<.001**
	L2	1.19	0.65	.066	24.47	7.35	**.001**	1.75	0.63	**.005**	17.34	7.00	**.013**
	S2	−0.20	0.22	.372	5.31	0.88	**<.001**	−0.40	0.22	.073	4.92	0.87	**<.001**
NA intensity	L1	10.64	0.92	<.001	119.27	14.93	**<.001**	9.43	0.82	<.001	98.78	12.00	**<.001**
	S1	−0.26	0.12	**.024**	1.03	0.25	**<.001**	−0.29	0.12	**.022**	1.65	0.28	**<.001**
	L2	−0.29	0.50	.560	5.92	4.73	.211	−0.32	0.51	.529	15.47	4.74	**.001**
	S2	0.44	0.17	**.008**	2.20	0.50	**<.001**	0.67	0.17	**<.001**	2.88	0.53	**<.001**
PA variability	L1	10.54	0.73	<.001	68.13	13.26	**<.001**	10.54	0.73	<.001	68.13	13.26	**<.001**
	S1	−0.60	0.12	**<.001**	1.11	0.42	**.008**	−0.60	0.12	**<.001**	1.11	0.42	**.008**
	L2	0.53	0.64	.411	17.11	10.22	.094	0.53	0.64	.411	17.11	10.22	.094
	S2	0.31	0.17	.073	1.95	0.91	**.033**	0.31	0.17	.073	1.95	0.91	**.033**
NA variability	L1	10.18	0.69	<.001	51.63	8.59	**<.001**	8.78	0.55	<.001	31.17	5.55	**<.001**
	S1	−0.59	0.13	**<.001**	0.99	0.33	**.003**	−0.58	0.11	**<.001**	0.50	0.23	**.025**
	L2	−0.53	0.65	.419	9.63	8.00	.229	0.53	0.60	.379	9.22	6.95	.184
	S2	0.58	0.18	**.001**	1.48	0.57	**.009**	0.66	0.14	**<.001**	0.13	0.38	.729

*Note*: L1 reflects the general level of affect intensity or variability before the lockdown. S1 reflects the general changes during the whole study period. L2 reflects the immediate change in intensity or variability the week before the lockdown and the first week of the lockdown. S2 reflects the gradual changes during the lockdown weeks, above and beyond the S1. Significant effects (*p* < .05) are highlighted in bold.

Abbreviations: L1, level 1; L2, level 2, NA, negative affect; PA, positive affect; S1, slope 1; S2, slope 2.

More gradual changes in the affect intensity or variability during the lockdown period (S2) were significant in five models. For both adolescents and parents, negative affect intensity increased during the 7 weeks of the lockdown (*M*_S2_ = 0.44, SE = 0.17, *p* = .008 and *M*_S2_ = 0.67, SE = 0.17, *p* < .001, respectively). Furthermore, negative affect variability increased for both adolescents and parents (*M*_S2_ = 0.58, SE = 0.18*, p* = .001 and *M*_S2_ = 0.66, SE = 0.14, *p* < .001 respectively). Finally, for parents, positive affect variability increased during the lockdown (*M*_S2_ = 0.57, SE = 0.16, *p* < .001) (see Table 3, and Supporting Information: Table S2).

**Figure 3:**
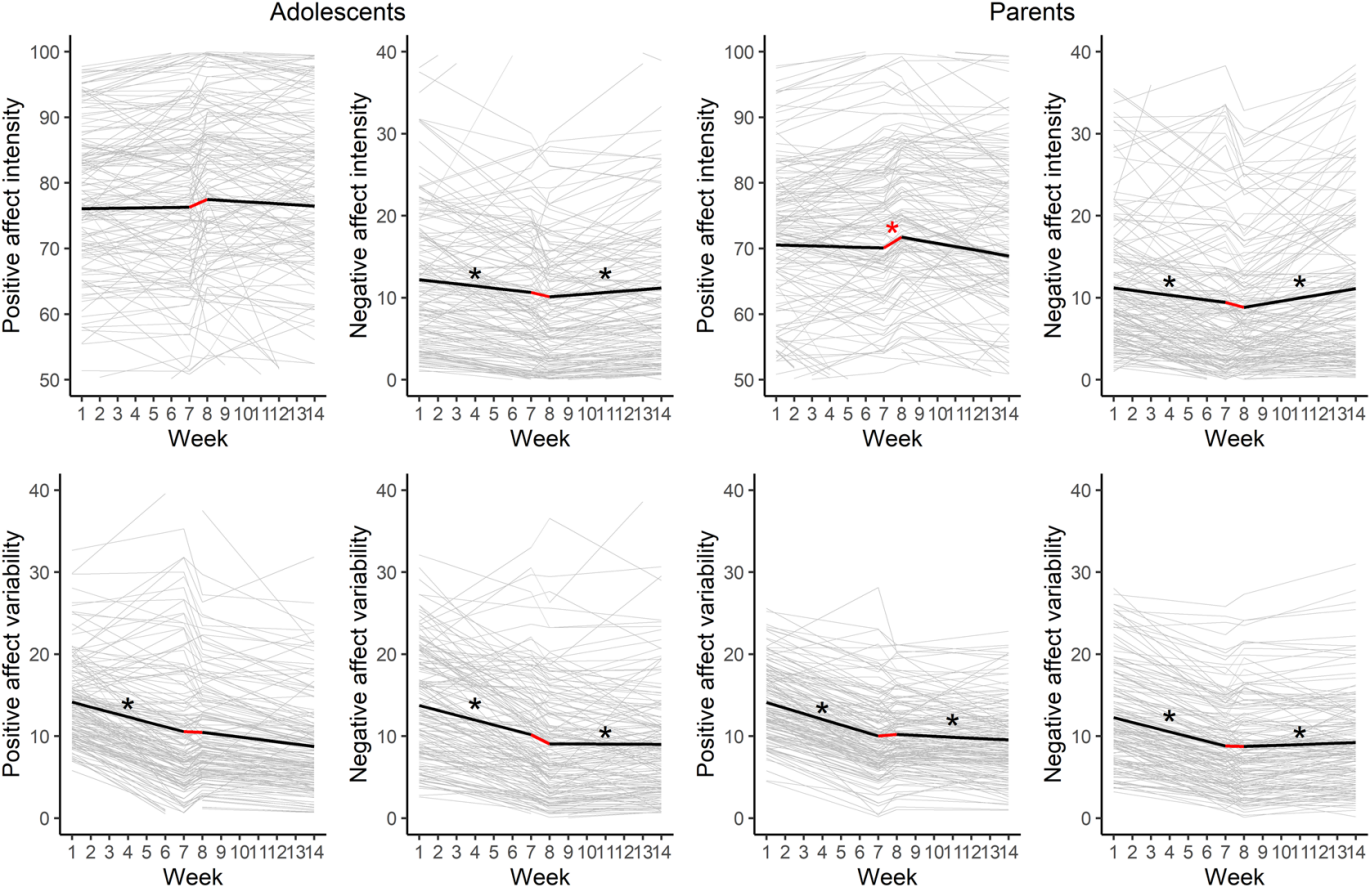
Piecewise growth models of positive and negative affect intensity and variability. The black line reflects the average estimated trajectory. The gray lines reflect the trajectories of the individual participants. The red star indicates the significant mean level change (L2), the black stars on the left of the red line indicate the significant overall slope (S1) and the black stars on the right of the red line indicate the significant slope during the lockdown weeks (S2). [Color figure can be viewed at wileyonlinelibrary.com]

Hence, even though the expected immediate effects were not found, and parents reported slightly more positive affect in the week after the lockdown introduction, both adolescents' and parents' level of negative affect and variability in negative affect increased gradually as the lockdown prolonged (Figure 3).

### 3.2 Aim 2: Age difference in the effect of the lockdown on affect intensity and variability

To assess whether adolescents and parents differ in the immediate (L2) or gradual changes (S2), we constrained these growth factors to be equal across age groups (see Supporting Information: Table S3). Not in line with our hypotheses, no significant differences were found between adolescents and parents in the lockdown effects on affect intensity, nor variability (H2). Adolescents and parents were, on average, affected in similar ways by the lockdown.

### 3.3 Aim 3: Individual differences in effects

**Figure 4:**
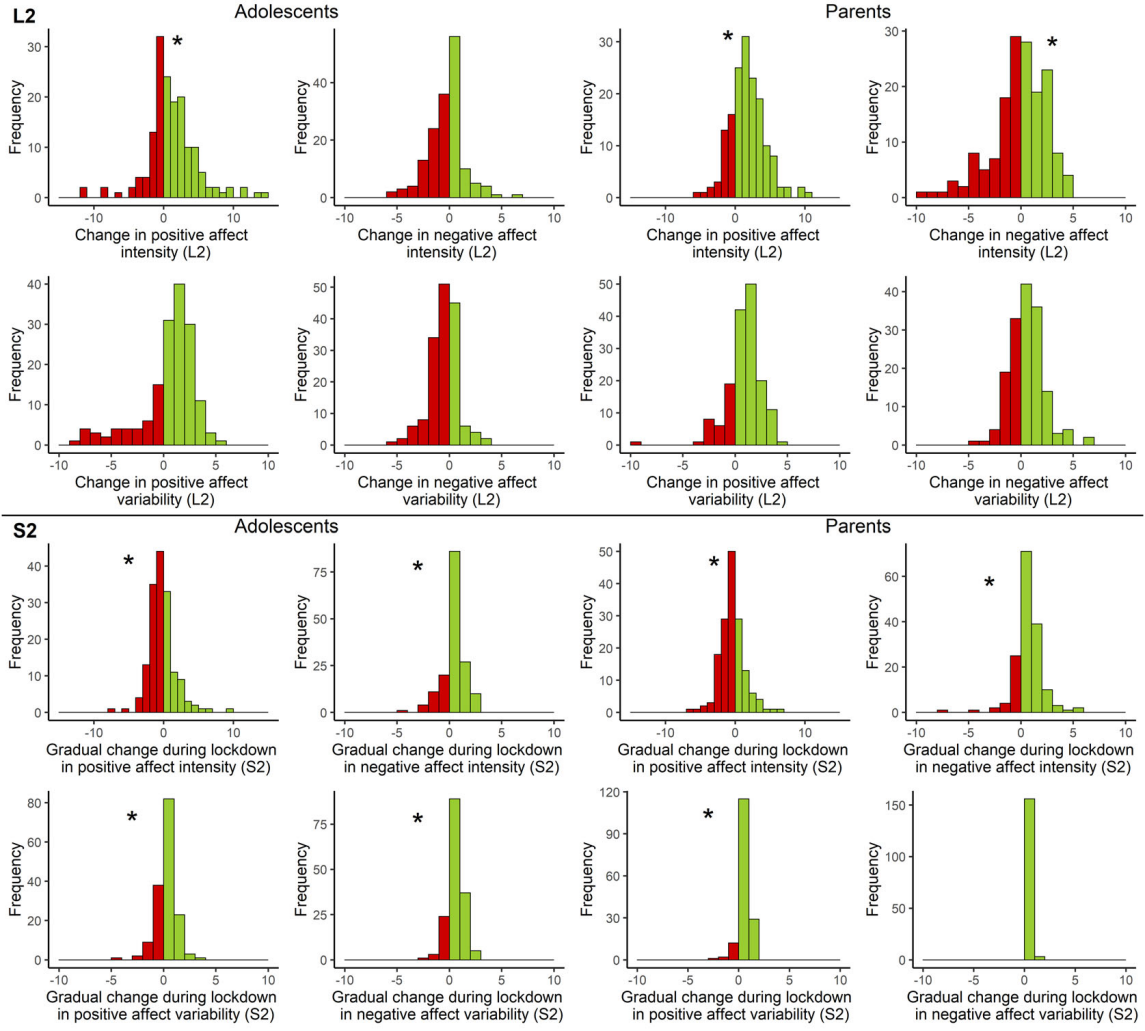
Individual differences in immediate (L2: upper panel) and gradual (S2: lower panel) change in affect intensity or variability during the weeks of the lockdown, for adolescents (left part) and their parents (right part). Stars indicate significant variance and thus individual differences in effects (*p* < .05). [Color figure can be viewed at wileyonlinelibrary.com]

As expected individuals differed in the immediate changes in affect intensity (H3) in three out of the eight models (Figure 4—variance around L2). The effect on adolescents' (Var_L2_ = 24.5, SE = 7.35, *p* = .001) and parents' positive affect intensity (Var_L2_ = 17.3, SE = 7.0, *p* = .013), and parents' negative affect intensity (Var_L2_ = 15.5, SE = 4.7, *p* = .001) differed significantly among participants (see Table 3). For instance, plotting these change rates per individual (upper panel Figure 4) demonstrated that some participants felt better at the lockdown start compared to the week before (i.e., decrease in negative affect intensity, increase in positive affect intensity), for others the intensity was stable, and for others well‐being decreased (i.e., increase in negative affect intensity and decrease in positive affect intensity).

Exploring whether individuals differed in the gradual changes during 7 weeks of the lockdown (S2), the variance was significant in seven of the eight models (except for negative affect variability for parents). The lower panel of Figure 4 shows that whereas for some participants the positive or negative affect intensity or variability gradually increased, it was stable or gradually decreased for others.

### 3.4 Aim 4: Individual differences and baseline life satisfaction and depressive symptoms

To better understand why some participants were more resilient against lockdown effects, we examined whether changes in intensity and variability would depend on baseline life satisfaction (adolescent models) or depressive symptoms (adolescent and parent models) (see Table 4 for the results and Supporting Information: Table S5 for model fit statistics). First, overall levels of affective well‐being (L1) were related to depressive symptoms and life satisfaction as expected (H4a): Adolescents and parents with fewer depressive symptoms and adolescents with higher life satisfaction reported higher positive affect intensity and lower negative affect intensity, and adolescents with more depressive symptoms had higher positive and negative affect variability (see Table 4). Contrary to our expectations (H4b), depressive symptoms or life satisfaction were unrelated to immediate lockdown effects (L2). However, gradual changes in positive affect during the lockdown period (S2) were stronger for adolescents with higher baseline life satisfaction (*M*_S2_ = 0.40, SE = 0.16, *p* = .011), that is, their positive affect decreased less strongly during the lockdown. Additionally, parents with more depressive symptoms experienced stronger increases in negative affect intensity during the lockdown (*M*_S2_ = 0.09, SE = 0.03, *p* = .002). In sum, even though depressive symptoms and life satisfactions were related to everyday experienced intensity and variability in emotional functioning over 100 days, they explained more of the gradual impact of the lockdown than the immediate impact.

**Table 4:** Effect of baseline well‐being, depressive symptoms, and self‐reported impact on the intercepts and slopes

		L1			S1			L2			S2		
		*M*	SE	*p*	*M*	SE	*p*	*M*	SE	*p*	*M*	SE	*p*
Adolescents													
PA intensity	LS	5.67	0.94	**<.001**	−0.17	0.1	.073	−0.32	0.46	.487	0.4	0.16	**.011**
NA intensity	LS	−3.15	0.62	**<.001**	0.1	0.08	.239	−0.35	0.36	.329	−0.06	0.12	.644
PA variability	LS	−1.13	0.61	.061	0.17	0.12	.148	−0.25	0.43	.558	−0.13	0.18	.455
NA variability	LS	−0.94	0.49	.055	0.3	0.09	**.001**	−0.9	0.46	.051	−0.17	0.13	.175
PA intensity	Depr	−1.32	0.2	**<.001**	0.03	0.02	.186	0.07	0.10	.467	−0.07	0.03	.041
NA intensity	Depr	0.74	0.13	**<.001**	−0.03	0.02	.147	0.06	0.08	.432	0.04	0.03	.122
PA variability	Depr	0.43	0.13	**.001**	0.00	0.02	.877	0.00	0.10	.985	−0.03	0.03	.377
NA variability	Depr	0.39	0.10	**<.001**	−0.06	0.02	**.006**	0.05	0.10	.637	0.06	0.03	.047
PA intensity	Impact	0.28	0.27	.308	−0.05	0.03	*.038*	0.25	0.12	*.033*	0.07	0.04	.094
NA intensity	Impact	−0.05	0.17	.767	0.03	0.02	.124	−0.16	0.09	.074	−0.04	0.03	248
PA variability	Impact	0.05	0.20	.785	0.03	0.03	.200	−0.14	0.16	.370	−0.03	0.04	.401
NA variability	Impact	0.07	0.13	.592	0.04	0.02	.115	−0.16	0.12	.173	−0.04	0.03	.249
Parents													
PA intensity	Depr	−1.56	0.22	**<.001**	−0.03	0.03	.271	0.1	0.11	.374	0.00	0.04	.999
NA intensity	Depr	0.65	0.14	**<.001**	−0.05	0.02	.041	‐0.01	0.09	.902	0.09	0.03	**.002**
PA variability	Depr	0.31	0.10	**.002**	−0.03	0.02	.147	0.08	0.11	.479	0.04	0.03	.202
NA variability	Depr	0.48	0.09	**<.001**	−0.05	0.02	**.009**	0.15	0.11	.157	0.03	0.03	.202
PA intensity	Impact	1.27	0.29	**<.001**	0.05	0.03	.147	0.19	0.13	.137	0.00	0.05	.974
NA intensity	Impact	−0.23	0.18	.208	0.06	0.03	*.022*	‐0.16	0.11	.142	‐0.08	0.04	*.022*
PA variability	Impact	0.10	0.12	.405	0.06	0.03	*.013*	‐0.28	0.13	*.036*	‐0.03	0.03	.447
NA variability	Impact	−0.11	0.12	.377	0.03	0.02	.184	‐0.15	0.13	.262	0.01	0.03	.807

*Note*: L1 reflects the general level of affect intensity or variability before the lockdown. S1 reflects the general changes during the whole study period. L2 reflects the immediate change in intensity or variability the week before the lockdown and the first week of the lockdown. S2 reflects the gradual changes during the lockdown weeks, above and beyond the S1. Significant effects (*p* < .05) are highlighted in bold.

Abbreviations: Depr, level of depressive symptoms; Impact, self‐reported impact of the lockdown on daily life and well‐being; L1, level 1; L2, level 2; LS, life satisfaction; NA, negative affect; PA, positive affect; S1, slope 1; S2, slope 2.

### 3.5 Exploratory analysis: Individual differences and lockdown impact and coping

Exploring individual differences in daily affect intensity and variability in relation to the self‐reported impact of the lockdown on daily life showed small effects (see Table 4). Parents with a higher level of positive affect (L1) reported a more positive influence of the lockdown on daily life (*M*_change_ = 1.27, SE = 0.29, *p* < .001). The other smaller effects between the self‐reported impact of the lockdown and changes in positive affect intensity and variability should be interpreted cautiously, since they do not reach significance when correcting for multiple testing. In sum, self‐reported impact of the lockdown on daily life explained small parts of the variance in experienced affect intensity and variability due to the lockdown.

We found a few effects of coping strategies on general positive and negative affect, that is, confrontation and social support were related to higher general positive affect intensity. However, the coping strategies were mostly unrelated to the lockdown effects on affect intensity and affect variability (see Supporting Information: Table S7). The only moderation effect was for confrontation on positive affect variability in the adolescent group (*M* = −0.64, SE = 0.18, *p* < .001). Adolescents who apply confrontation more often in response to difficulty or problems showed a stronger decrease in positive affect variability directly after the introduction of the lockdown.

## 4 DISCUSSION

The COVID‐19 pandemic and resulting lockdowns has a large impact on people's daily life, and emotional well‐being (Masten & Motti‐Stefanidi, 2020; Prati & Mancini, 2021; Robinson et al., 2022), and it is unsure whether future lockdowns are needed. By investigating the effect of the second lockdown in the Netherlands on daily affect of adolescents and their parents in more detail, this study aimed to understand the specific effects of a second lockdown, instead of the effects of first lockdowns, in which the acute response to the pandemic and experienced uncertainty about the virus were combined.

Using 100 days of diary data, the average adolescent and parent experienced hardly any immediate lockdown effects on affect intensity and variability. Unexpectedly, parents reported more positive affect in the week after the lockdown introduction. However, as the lockdown prolonged and both parents and adolescents were not allowed to sport, go to school or work, and had limited contact with friends and family, the intensity of negative affect and variability in positive and negative affect increased gradually. Moreover, although adolescents and parents were affected in similar ways, there was large heterogeneity between individuals. That is, some participants experienced a gradual increase in negative affect intensity and variability and decrease in positive affect intensity, whereas others remained stable or showed opposite patterns. Baseline depressive symptoms and life satisfaction partly explained individual differences in these effects, with baseline life satisfaction promoting positive affect, and depressive symptoms related to larger increases in negative affect during the lockdown. Furthermore, individuals who reported a larger lockdown impact on daily life showed slightly stronger lockdown effects on affect intensity and variability.

### 4.1 Changes in daily affect intensity and variability during the lockdown

Based on extensive earlier work, we expected that the lockdown would decrease well‐being immediately, as operationalized by change in daily affect intensity and variability. However, the only immediate effect we found was an *increase* in positive affect intensity of parents in first lockdown week compared to the week before the lockdown. An explanation might be relief of parents over clarity about the restrictions. In the weeks leading up to the lockdown, the number of COVID‐19 cases increased steadily, and people started to expect a lockdown any time soon, leading to uncertainty about the situation and perhaps distress and anxiousness (Reizer et al., 2021). The absence of other immediate effects might be explained by the expectation and anticipation of an upcoming lockdown (Brodeur et al., 2021). Anticipation could have prepared people mentally, reducing direct effects of the lockdown when introduced.

Even though at the start of the second lockdown, adolescents and parents were not much affected, as the lockdown endured, negative effects on daily affect emerged. The gradual decreases in positive affect intensity and increases in negative affect intensity are in line with other longitudinal studies (Brodeur et al., 2021; Pellerin & Raufaste, 2020). However, in contrast to our expected decrease in positive affect variability (Deng et al., 2021; Green et al., 2021), we report a gradual increase in positive and negative affect variability during the lockdown. Whereas earlier studies compared affect variability at two time points (i.e., prelockdown vs. lockdown), our findings add insights about gradual and subtle increases in affect variability during the lockdown. Affect variability is useful within boundaries, but the gradual increase in positive and negative variability can be a risk factor for future emotional problems.

The gradual changes implicate the need to monitor people's well‐being longitudinally during a lockdown to not miss gradually emerging adverse effects when the lockdown prolongs. As indicated by Robinson et al. (2022), on average people returned to their baseline well‐being relatively quickly after the first lockdown ended. However, as individual differences in this effect are found, part of the population does not restore their well‐being and are at risk for psychopathology, such as depressive symptoms or anxiety (Galatzer‐Levy et al., 2018). Psychopathology symptoms do not arise overnight, but through the accumulation of negative events and feelings. Therefore, to reliably figure out the effects of later lockdowns on well‐being, both short‐term and longer‐term effects during the lockdown and after the lockdown ends should be monitored, especially in groups at risk.

### 4.2 Differences between adolescents and parents

There were several reasons (i.e., different restrictions, emotional development, and stressful developmental period) why we expected adolescents to be more influenced by the lockdown compared to their parents. Yet, no such differences were found. On average, parents and adolescents in this study responded in quite similar ways. An explanation of this absence of differences might be, contrary to the expectation, that, on average, parents indicated a larger negative self‐reported lockdown effect on daily life (*M* = −3.80, SD = 4.63) compared to adolescents (*M* = −1.16, SD = 5.51, *t* = 5.29, *p* < .001). Although the actual transition from pre‐lockdown to lockdown in terms of regulations was objectively larger for adolescents than adults, parents felt more restricted. In line with this idea, in the first lockdown, Janssen et al. (2020) reported that parents, but not adolescents increased in their negative affect in two‐week period during the COVID‐19 pandemic compared to pre‐pandemic.

The direct comparison of the lockdown effects for adolescents and their own parents is a strong test, since it reduces random factors that would be there if adolescents and parents were from different households. The adolescent and parent belong to the same family, live in the same household, and experience similar stressors. They also share part of their genetic predispositions for well‐being and risk for psychopathology (Bartels et al., 2013; Silberg et al., 2010). This within‐household design and shared environmental factors might explain the absence of differences between adolescents and their parents. Furthermore, the affect of adolescents and their parents is related and adolescents can influence parents' affect and vice versa (Griffith et al., 2021; Kim et al., 2001). We report a positive (within‐family) correlation between positive affect intensity of adolescents and their parents (see Supporting Information: Table S10), suggesting this mutual influence and synchrony between affect of parents and adolescents (see the temporal interpersonal emotion systems [“TIES”] model, Butler, 2011; Lougheed & Keskin, 2021).

In conclusion, the similar lockdown effects on adolescents and their parents could be because parents and children share the same environment and influence each other. However, this does not necessarily indicate that the underlying mechanism contributing to the negative effects of the pandemic on well‐being is the same for adolescents and adults. We hypothesized that the prolonged stress and restriction led to decrease in affective well‐being, with a larger effect for adolescents, because of missing out on opportunities for social development and growth. However, future research is needed to investigate the specific underlying mechanisms.

### 4.3 Individual differences

This study demonstrated heterogeneity in individuals' responses to a lockdown. Whereas some participants showed the expected effects, that is, gradual increases in negative affect intensity and variability and decreases in positive affect, others were stable or showed opposite patterns. People differ in the effects, which can be explained by different environmental factors and stressors experienced in the lockdown, and genetic predispositions for well‐being, and sensitivity to extreme environmental changes due to the pandemic (de Vries et al., 2021; Rimfeld et al., 2021; van de Weijer et al., 2022).

To further understand individual differences in the effects, we investigated the association with baseline life satisfaction and depressive symptoms. The results led to insights about the protective effects of general well‐being measures in extreme environmental situations, that is, the lockdown, on daily affective well‐being. As expected (Aan het Rot et al., 2012; Houben et al., 2015; Reitsema et al., 2022), higher affect variability was associated with more depressive symptoms and lower life satisfaction. However, they only explained small parts of the differential responses to the lockdown. Among adolescents, those with higher baseline life satisfaction reported a stronger increase in positive affect during the lockdown. Similarly, for parents, those who reported more depressive symptoms experienced a stronger gradual increase in negative affect during the lockdown. If replicated, these effects could indicate more resilience to negative lockdown effects for people with a higher life satisfaction or fewer depressive symptoms. Based on the resilience theory that individuals who can cope better with stressful events are less affected by the lockdown, we investigated the association of the lockdown effects with four different coping strategies, confrontation, avoidance, social support and palliative reaction. However, these coping strategies were mostly unrelated to the lockdown effects on affect intensity and affect variability.

The specific lockdown effects on people's daily life and environment differed considerably across people and this can be an explanation for individual differences in the lockdown effects on affect. For example, some parents had to work from home, whereas others continued working on site. Similarly, some adolescents might not have their own room and argue more with siblings. Moreover, some people could be more sensitive to their environment than others, and therefore, more strongly affected by lockdown restrictions and accompanied family stress (Greven et al., 2019; Pluess, 2015). To capture these individual differences in the impact of the lockdown on daily life, we explored the associations between the self‐reported impact of the lockdown of the lockdown on daily life and well‐being and the lockdown effects on affect intensity and variability. We found small associations. Individuals who reported a negative effect of the lockdown on daily life and well‐being also showed stronger lockdown effects on daily affect intensity and variability. However, these results should be interpreted with caution, since both the daily affect and lockdown impact on daily life was based on self‐reports and the effects can emerge due to the common method bias (Podsakoff et al., 2003). Furthermore, Janssen et al. (2020) reported that pandemic related characteristics (i.e., the working from home, children present at home etc.) could not explain the individual differences in positive and negative affect during the first lockdown in their sample. Therefore, more research to the causes of individual differences in response to the lockdown or any other word‐wide change are needed. For example, differences in response to (environmental) change are driven by differences in genetic background between people. Therefore gene–environment interaction and gene–environmental correlations should be taken into account in future studies either by using genetically informative designs, such as twin studies, or by adding polygenic scores to the models.

### 4.4 Future directions and limitations

Although the unique data set with 100 days of data per family was sufficiently powered to answer our preregistered hypotheses, there are some limitations to the current study. A first limitation is the sample representativeness. In both the adolescent and parent group, there was an overrepresentation of females (62% and 80%, respectively). Additionally, compared to the general Dutch population, in which ~35% of the adults attended higher vocational school or university and 45% of the adolescents currently follow higher secondary education (Statistics Netherlands, 2020), the current sample is highly educated (adults: 63%, adolescents: 80%). More research to lockdown effects on daily affect in diverse samples and participants from lower socioeconomic backgrounds is needed to generalize results to the whole population.

Furthermore, a possible confounding factor is the timing of the second lockdown in the Netherlands. The lockdown started in the middle of December (December 15) and lasted until the end of February, meaning a lockdown during the winter holidays. The ritual of celebrating Christmas can lead to increased well‐being or, when experienced as a stressor, to conflict and lower well‐being and higher negative affect (Mutz, 2016; Páez et al., 2011). Furthermore, the start of vaccination program was announced in December in the Netherlands, leading to hope. Therefore, the overlap of different events could have influenced the results.

Additionally, the results of this Dutch study might be difficult to generalize to other countries. Although the COVID‐19 virus is a global pandemic, countries are differently affected by the pandemic and governments implemented necessary restrictions at different times. Especially relevant for later waves of the virus, the pace of vaccination and willingness of people to be vaccinated created diverse situations in countries.

Finally, we assessed a few potential factors that could explain individual differences. Future studies should investigate possible moderating effects of the situation and individual differences in genetic sensitivity to extreme environmental change on the lockdowns effects on daily affect. Also, individual differences in personality might play a role in the experience of the lockdown situation (Kroencke et al., 2020; Modersitzki et al., 2021), for example, some adolescents may enjoy spending more time at home. More knowledge of the causes of heterogeneity in the effects is needed to increase resilience to lockdown effects in the population.

## 5 CONCLUSION

This 100‐day daily diaries study aimed to understand how everyday well‐being of adolescents and parents was affected by the second COVID‐19 lockdown, in which physical activity, social contact, and regular school and work patterns were disrupted. The daily affect of Dutch adolescents and their parents was, on average, not much affected immediately after the start of the lockdown. The only significant change was an increase in positive affect for parents. However, as the lockdown prolonged, more negative effects of lockdowns emerged both for parents and for adolescents, which have also been described in other studies. Specifically, negative affect intensity and variability increased and positive affect intensity decreased. However, the extent to which well‐being was impacted varied significantly between individuals, with parents who already felt depressed appeared at increased risk, and adolescents with higher baseline satisfaction at lower risk for negative impacts. Hence, when policy‐makers consider to use lockdowns as a means to combat the virus, these gradually emerging emotional costs for specific target groups should also be considered.

## Supporting information

Supporting information.

## Data Availability

The data that support the findings of this study are available from the corresponding author upon reasonable request.
