# Supplementary material for: Daily affect intensity and variability of adolescents and their parents before and during a COVID‐19 lockdown
Source: J Adolesc. 2022 Nov 7;95(2):336–53. doi: 10.1002/jad.12117 (PMC10100109; doi:10.1002/jad.12117)
Supplement: Supplementary file 1 — Supporting information. [file JAD-95-336-s001.docx]

**Supplementary material**

**Sensitivity analyses**

As a sensitivity analysis, we computed the positive and negative affect intensity and variability separately for the 50 days before the lockdown and the 50 days in the lockdown. Using a repeated measures test and including the two groups, we directly compared the affect intensity and variability of adolescents and their parents, the effect of the lockdown (pre-lockdown vs lockdown) and the interaction between the group and lockdown on positive and negative affect intensity and variability. This provides another test on whether adolescents and their parents reacted differently to the lockdown in terms of affect dynamics. Adolescents reported higher positive affect intensity compared to their parents, during the whole study period (*p*<.001), i.e., a difference between L1 in the main study. There are no differences between adolescents and their parents in negative affect intensity and positive and negative affect variability. Furthermore, the interaction effects between group and the lockdown period were not significant (see supplementary Table S4).

As another sensitivity analysis, we ran the main models with stricter thresholds for data inclusion. If a participant had more than 2 missing values in a week (i.e. less than 5 assessments in a week), the daily affect and variability scores were not computed for that specific week and not included in the models (resulting in the removal of 227 (10.2% of all available weeks) and 118 (5.3% of all available weeks) weeks for adolescents and parents respectively. Stricter inclusion thresholds did not affect the results (see supplementary Table S8 and S9). Therefore, the results with the larger groups are reported.

**Within-family effects**

As exploratory analysis, we correlated the estimated intercepts and slopes of the adolescents to the estimates of their parents. There were two significant correlations (see supplementary Table S10). We found a positive significant correlation between the general positive affect intensity (L1) of adolescent and parent before the lockdown (*r*=.27, *p*<.001), indicating that daily positive affect intensity between the adolescent and their parent is related. Furthermore, we found a significant positive correlation between the effect of the lockdown on the negative affect variability (L2) of adolescent and parent (*r*=.20, *p*=.011). If an adolescent showed an increased negative affect variability during the first lockdown week compared to the week before the lockdown, the same pattern was likely to occur for their parent.

**Supplementary figure**

**Figure S1.** Example of low and high positive affect intensity (top row) and low and high affect variability (bottom row).


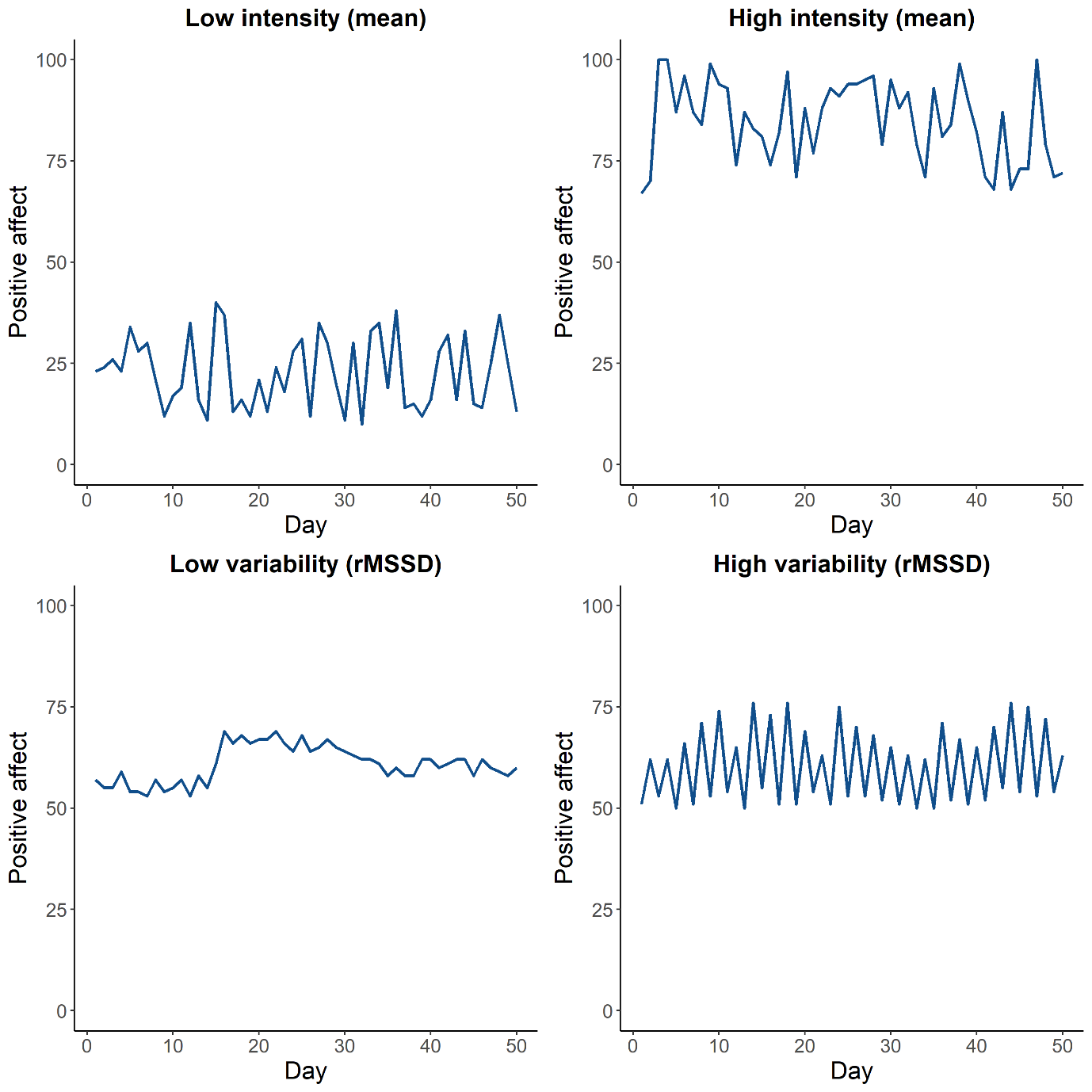


**Supplementary tables**

**Table S1.** Model fit of main models.

|  | **Adolescents** | | | | **Parents** | | | | |
| --- | --- | --- | --- | --- | --- | --- | --- | --- | --- |
|  | **RMSEA** | **CFI** | **TLI** |  | | **RMSEA** | **CFI** | **TLI** |  |
| PA intensity | 0.095 (.08-.11) | 0.938 | 0.938 | Acceptable | | 0.101 (.09-.12) | 0.960 | 0.960 | Acceptable |
| NA intensity | 0.088 (.07-.10) | 0.924 | 0.924 | Acceptable | | 0.096 (.08-.11) | 0.946 | 0.946 | Acceptable |
| PA variability | 0.058 (.04-.08) | 0.909 | 0.909 | Acceptable | | 0.041 (.01-.06) | 0.958 | 0.958 | Acceptable |
| NA variability | 0.035 (.00-.06) | 0.972 | 0.972 | Acceptable | | 0.070 (.05-.09) | 0.922 | 0.922 | Acceptable |

*Note*: PA= positive affect, NA = negative affect. RMSEA= root mean square error of approximation, CFI= comparative fit index, TLI= Tucker-Lewis index. Model fit is estimated with maximum likelihood with robust standard errors (MLR).

**Table S2.** Correlations between the intercepts and slopes.

| **Adolescents** | | | | | | | | | |
| --- | --- | --- | --- | --- | --- | --- | --- | --- | --- |
| PA intensity | L1 | S1 | L2 | S2 | NA intensity | L1 | S1 | L2 | S2 |
| L1 | 1 |  |  |  | L1 | 1 |  |  |  |
| S1 | **0.52** | 1 |  |  | S1 | **0.48** | 1 |  |  |
| L2 | -0.27 | 0.28 | 1 |  | L2 | -0.29 | 0.19 | 1 |  |
| S2 | **-0.42** | **-0.84** | -0.07 | 1 | S2 | **-0.48** | **-0.84** | 0.33 | 1 |
| PA variability | L1 | S1 | L2 | S2 | NA variability | L1 | S1 | L2 | S2 |
| L1 | 1 |  |  |  | L1 | 1 |  |  |  |
| S1 | *0.78* | 1 |  |  | S1 | 0.33 | 1 |  |  |
| L2 | *-0.85* | -0.75 | 1 |  | L2 | -0.26 | -0.41 | 1 |  |
| S2 | *-0.79* | *-0.89* | 0.66 | 1 | S2 | *-0.46* | *-0.68* | -0.14 | 1 |
| **Parents** | | | | | | | | | |
| PA intensity | L1 | S1 | L2 | S2 | NA intensity | L1 | S1 | L2 | S2 |
| L1 | 1 |  |  |  | L1 | 1 |  |  |  |
| S1 | **0.66** | 1 |  |  | S1 | **0.59** | 1 |  |  |
| L2 | *-0.59* | *-0.59* | 1 |  | L2 | **-0.70** | **-0.96** | 1 |  |
| S2 | **-0.50** | **-0.80** | 0.42 | 1 | S2 | **-0.40** | **-0.77** | *0.61* | 1 |
| PA variability | L1 | S1 | L2 | S2 | NA variability | L1 | S1 | L2 | S2 |
| L1 | 1 |  |  |  | L1 | 1 |  |  |  |
| S1 | *0.47* | 1 |  |  | S1 | -0.03 | 1 |  |  |
| L2 | -0.55 | -0.80 | 1 |  | L2 | 0.22 | -0.94 | 1 |  |
| S2 | *-0.50* | *-0.74* | 0.73 | 1 | S2 | -0.03 | -0.64 | 0.81 | 1 |

*Note.* bold correlations are significant at p<.001, italic correlations are significant at p<.05. PA= positive affect, NA = negative affect. L1= level 1, L2 = level 2, S1= slope 1, S2= slope 2.

**Table S3.** Results of the model comparisons when constrain the estimates of the parent models to be equal to the adolescent models.

|  |  | **Chi square** | **df** | **Δchi square** | **Δdf** | **p-value** |
| --- | --- | --- | --- | --- | --- | --- |
| PA intensity | Parent model | 237.18 | 91 |  |  |  |
|  | L2 constrained | 237.98 | 92 | 0.81 | 1 | 0.370 |
|  | S2 constrained | 237.98 | 92 | 0.80 | 1 | 0.371 |
| NA intensity | Parent model | 224.73 | 91 |  |  |  |
|  | L2 constrained | 224.73 | 92 | 0.01 | 1 | 0.994 |
|  | S2 constrained | 226.52 | 92 | 1.79 | 1 | 0.181 |
| PA variability | Parent model | 115.38 | 91 |  |  |  |
|  | L2 constrained | 115.69 | 92 | 0.31 | 1 | 0.565 |
|  | S2 constrained | 118.08 | 92 | 2.70 | 1 | 0.100 |
| NA variability | Parent model | 162.38 | 91 |  |  |  |
|  | L2 constrained | 165.45 | 92 | 3.06 | 1 | 0.080 |
|  | S2 constrained | 162.70 | 92 | 0.31 | 1 | 0.576 |

*Note.* The submodels with L2 or S2 constrained are compared to the parent model. PA= positive affect, NA = negative affect. L2 = level 2, S2= slope 2.

**Table S4**. Results of the repeated measures ANOVA (pre- vs lockdown) with group (adolescent vs adult) included.

| **PA intensity** | *β* | *SE* | *p* | **NA intensity** | *β* | *SE* | *p* |
| --- | --- | --- | --- | --- | --- | --- | --- |
| Intercept | 77.22 | 1.29 | **<2x10^-16^** | Intercept | 10.64 | 0.78 | **<2x10^-16^** |
| Lockdown | -1.09 | 0.58 | 0.062 | Lockdown | 0.91 | 0.42 | 0.031 |
| Group | -7.13 | 1.83 | **1.17x10^-04^** | Group | -0.39 | 1.10 | 0.720 |
| LD*group | 1.27 | 0.82 | 0.122 | LD*group | -0.80 | 0.59 | 0.180 |
| **PA variability** | *β* | *SE* | *p* | **NA variability** | *β* | *SE* | *p* |
| Intercept | 11.23 | 0.53 | **<2x10^-16^** | Intercept | 11.88 | 0.62 | **<2x10^-16^** |
| Lockdown | 3.30 | 0.46 | **4.15x10^-12^** | Lockdown | 3.09 | 0.53 | **1.16x10^-08^** |
| Group | 0.27 | 0.75 | 0.72 | Group | -0.14 | 0.87 | 0.876 |
| LD*group | -1.01 | 0.65 | 0.122 | LD*group | -1.52 | 0.74 | 0.043 |

*Note*. LD= lockdown. PA= positive affect, NA = negative affect.

**Table S5.** Model fit covariate models.

| **Adolescents** | |  | |  |  |  |
| --- | --- | --- | --- | --- | --- | --- |
| Depressive symptoms | | **RMSEA** | | **CFI** | **TLI** |  |
|  | PA intensity | | 0.126 (.11-.14) | 0.934 | 0.931 | Acceptable |
|  | NA intensity | | 0.107 (.09-.12) | 0.934 | 0.932 | Acceptable |
|  | PA variability | | 0.055 (.04-.07) | 0.916 | 0.912 | Acceptable |
|  | NA variability | | 0.060 (.04-.08) | 0.947 | 0.945 | Acceptable |
| Well-being | | **RMSEA** | | **CFI** | **TLI** |  |
|  | PA intensity | | 0.127 (.11-.14) | 0.933 | 0.930 | Acceptable |
|  | NA intensity | | 0.105 (.09-.12) | 0.936 | 0.934 | Acceptable |
|  | PA variability | | 0.056 (.04-.07) | 0.912 | 0.908 | Acceptable |
|  | NA variability | | 0.062 (.04-.08) | 0.942 | 0.939 | Acceptable |
| Impact | | **RMSEA** | | **CFI** | **TLI** |  |
|  | PA intensity | | 0.132 (.12-.15) | 0.928 | 0.926 | Acceptable |
|  | NA intensity | | 0.105 (.09-.12) | 0.938 | 0.936 | Acceptable |
|  | PA variability | | 0.055 (.03-.07) | 0.912 | 0.909 | Acceptable |
|  | NA variability | | 0.066 (.05-.08) | 0.930 | 0.927 | Acceptable |
| **Parents** | |  | |  |  |  |
| Depressive symptoms | | **RMSEA** | | **CFI** | **TLI** |  |
|  | PA intensity | | 0.096 (.08-.11) | 0.960 | 0.959 | Acceptable |
|  | NA intensity | | 0.097 (.08-.11) | 0.940 | 0.938 | Acceptable |
|  | PA variability | | 0.048 (.02-.07) | 0.945 | 0.943 | Acceptable |
|  | NA variability | | 0.069 (.05-.09) | 0.923 | 0.920 | Acceptable |
| Impact | | **RMSEA** | | **CFI** | **TLI** |  |
|  | PA intensity | | 0.097 (.08-.11) | 0.960 | 0.958 | Acceptable |
|  | NA intensity | | 0.093 (.08-.11) | 0.944 | 0.942 | Acceptable |
|  | PA variability | | 0.046 (.02-.07) | 0.947 | 0.945 | Acceptable |
|  | NA variability | | 0.066 (.05-.08) | 0.924 | 0.921 | Acceptable |

Note: PA= positive affect, NA = negative affect. RMSEA= root mean square error of approximation, CFI= comparative fit index, TLI= Tucker-Lewis index.

**Table S6.** Model fit covariate models coping strategies.

| **Adolescents** | |  | |  |  |  |
| --- | --- | --- | --- | --- | --- | --- |
| Confrontation | | **RMSEA** | | **CFI** | **TLI** |  |
|  | PA intensity | | 0.130 (.12-.15) | .931 | .928 | Acceptable |
|  | NA intensity | | 0.103 (.09-.12) | .940 | .938 | Acceptable |
|  | PA variability | | 0.050 (.03-.07) | .929 | .926 | Acceptable |
|  | NA variability | | 0.062 (.04-.08) | .937 | .934 | Acceptable |
| Avoidance | | **RMSEA** | | **CFI** | **TLI** |  |
|  | PA intensity | | 0.132 (.12-.15) | .929 | .926 | Acceptable |
|  | NA intensity | | 0.103 (.09-.12) | .940 | .938 | Acceptable |
|  | PA variability | | 0.053 (.03-.07) | .921 | .918 | Acceptable |
|  | NA variability | | 0.061 (.04-.08) | .940 | .938 | Acceptable |
| Social support | | **RMSEA** | | **CFI** | **TLI** |  |
|  | PA intensity | | 0.133 (.12-.15) | .928 | .925 | Acceptable |
|  | NA intensity | | 0.106 (.09-.12) | .938 | .935 | Acceptable |
|  | PA variability | | 0.057 (.04-.07) | .909 | .905 | Acceptable |
|  | NA variability | | 0.066 (.05-.08) | .929 | .926 | Acceptable |
| Palliative reaction | | | **RMSEA** | **CFI** | **TLI** |  |
|  | PA intensity | | 0.133 (.12-.15) | .927 | .925 | Acceptable |
|  | NA intensity | | 0.107 (.09-.12) | .937 | .934 | Acceptable |
|  | PA variability | | 0.050 (.03-.07) | .928 | .926 | Acceptable |
|  | NA variability | | 0.060 (.04-.08) | .941 | .938 | Acceptable |
| **Parents** | |  | |  |  |  |
| Confrontation | | **RMSEA** | | **CFI** | **TLI** |  |
|  | PA intensity | | 0.097 (.08-.11) | .959 | .958 | Acceptable |
|  | NA intensity | | 0.090 (.08-.11) | .948 | .946 | Acceptable |
|  | PA variability | | 0.000 (.00-.04) | 1.00 | 1.00 | Acceptable |
|  | NA variability | | 0.068 (.05-.09) | .921 | .918 | Acceptable |
| Avoidance | | **RMSEA** | | **CFI** | **TLI** |  |
|  | PA intensity | | 0.097 (.08-.11) | .959 | .958 | Acceptable |
|  | NA intensity | | 0.093 (.08-.11) | .944 | .942 | Acceptable |
|  | PA variability | | 0.013 (.00-.04) | .993 | .993 | Acceptable |
|  | NA variability | | 0.068 (.05-.09) | .919 | .915 | Acceptable |
| Social support | | **RMSEA** | | **CFI** | **TLI** |  |
|  | PA intensity | | 0.096 (.08-.11) | .960 | .959 | Acceptable |
|  | NA intensity | | 0.098 (.08-.11) | .938 | .936 | Acceptable |
|  | PA variability | | 0.000 (.00-.04) | 1.00 | 1.00 | Acceptable |
|  | NA variability | | 0.075 (.06-.09) | .903 | .899 | Acceptable |
| Palliative reaction | | | **RMSEA** | **CFI** | **TLI** |  |
|  | PA intensity | | 0.097 (.08-.11) | .959 | .958 | Acceptable |
|  | NA intensity | | 0.095 (.08-.11) | .942 | .940 | Acceptable |
|  | PA variability | | 0.000 (.00-.03) | 1.00 | 1.00 | Acceptable |
|  | NA variability | | 0.069 (.05-.09) | .917 | .914 | Acceptable |

Note: PA= positive affect, NA = negative affect. RMSEA= root mean square error of approximation, CFI= comparative fit index, TLI= Tucker-Lewis index.

**Table S7.**

*Effect of coping strategies on the intercepts and slopes.*

|  | | **L1** |  |  | **S1** |  |  | **L2** |  |  | **S2** |  |  |
| --- | --- | --- | --- | --- | --- | --- | --- | --- | --- | --- | --- | --- | --- |
| **Adolescents** | | *M* | *SE* | *p* | *M* | *SE* | *p* | *M* | *SE* | *p* | *M* | *SE* | *p* |
| Confrontation | PA intensity | 1.26 | 0.45 | ***0.005*** | 0.01 | 0.04 | 0.797 | 0.02 | 0.20 | 0.932 | 0.00 | 0.07 | 0.973 |
|  | NA intensity | -0.33 | 0.29 | 0.264 | 0.03 | 0.04 | 0.444 | -0.02 | 0.16 | 0.887 | -0.06 | 0.05 | 0.232 |
|  | PA variability | 0.35 | 0.21 | 0.105 | 0.09 | 0.04 | ***0.010*** | -0.64 | 0.18 | **0.000** | -0.10 | 0.06 | 0.065 |
|  | NA variability | 0.18 | 0.21 | 0.409 | 0.04 | 0.04 | 0.283 | -0.18 | 0.20 | 0.368 | -0.07 | 0.05 | 0.195 |
|  | | *M* | *SE* | *p* | *M* | *SE* | *p* | *M* | *SE* | *p* | *M* | *SE* | *p* |
| Avoidance | PA intensity | -1.61 | 0.91 | 0.078 | -0.02 | 0.09 | 0.802 | -0.63 | 0.40 | 0.110 | 0.15 | 0.14 | 0.280 |
|  | NA intensity | 0.01 | 0.58 | 0.987 | -0.01 | 0.08 | 0.849 | 0.10 | 0.31 | 0.753 | 0.09 | 0.11 | 0.385 |
|  | PA variability | 0.16 | 0.47 | 0.727 | -0.03 | 0.08 | 0.670 | 0.41 | 0.38 | 0.282 | 0.04 | 0.11 | 0.701 |
|  | NA variability | 0.32 | 0.42 | 0.449 | 0.04 | 0.08 | 0.654 | -0.50 | 0.40 | 0.212 | 0.05 | 0.11 | 0.669 |
|  | | *M* | *SE* | *p* | *M* | *SE* | *p* | *M* | *SE* | *p* | *M* | *SE* | *p* |
| Social support | PA intensity | 1.00 | 0.34 | ***0.003*** | -0.03 | 0.03 | 0.429 | 0.09 | 0.15 | 0.550 | 0.07 | 0.05 | 0.190 |
|  | NA intensity | -0.26 | 0.22 | 0.235 | 0.01 | 0.03 | 0.867 | -0.11 | 0.12 | 0.340 | -0.04 | 0.04 | 0.331 |
|  | PA variability | -0.21 | 0.17 | 0.239 | 0.01 | 0.03 | 0.734 | -0.09 | 0.16 | 0.589 | 0.01 | 0.04 | 0.818 |
|  | NA variability | -0.05 | 0.16 | 0.773 | 0.01 | 0.03 | 0.796 | -0.03 | 0.15 | 0.871 | -0.02 | 0.04 | 0.584 |
|  | | *M* | *SE* | *p* | *M* | *SE* | *p* | *M* | *SE* | *p* | *M* | *SE* | *p* |
| Palliative reaction | PA intensity | 0.56 | 0.59 | 0.343 | 0.06 | 0.05 | 0.290 | -0.20 | 0.25 | 0.444 | -0.09 | 0.09 | 0.297 |
|  | NA intensity | 0.18 | 0.37 | 0.628 | 0.02 | 0.05 | 0.714 | -0.18 | 0.20 | 0.373 | 0.00 | 0.07 | 0.994 |
|  | PA variability | 0.14 | 0.29 | 0.634 | -0.03 | 0.05 | 0.513 | 0.11 | 0.24 | 0.641 | 0.05 | 0.07 | 0.452 |
|  | NA variability | 0.30 | 0.27 | 0.276 | -0.04 | 0.05 | 0.492 | -0.18 | 0.26 | 0.495 | 0.10 | 0.07 | 0.157 |
|  | | **L1** |  |  | **S1** |  |  | **L2** |  |  | **S2** |  |  |
| **Parents** | | *M* | *SE* | *p* | *M* | *SE* | *p* | *M* | *SE* | *p* | *M* | *SE* | *p* |
| Confrontation | PA intensity | 1.87 | 0.42 | **0.000** | 0.03 | 0.05 | 0.492 | -0.06 | 0.19 | 0.762 | 0.07 | 0.07 | 0.334 |
|  | NA intensity | -0.85 | 0.25 | **0.001** | 0.03 | 0.04 | 0.499 | 0.11 | 0.16 | 0.483 | -0.08 | 0.05 | 0.136 |
|  | PA variability | -0.31 | 0.22 | 0.160 | 0.02 | 0.04 | 0.696 | 0.21 | 0.22 | 0.337 | -0.08 | 0.06 | 0.178 |
|  | NA variability | -0.51 | 0.17 | ***0.003*** | 0.06 | 0.03 | 0.059 | 0.00 | 0.19 | 0.988 | -0.10 | 0.04 | 0.021 |
|  | | *M* | *SE* | *p* | *M* | *SE* | *p* | *M* | *SE* | *p* | *M* | *SE* | *p* |
| Avoidance | PA intensity | -2.15 | 0.93 | 0.021 | -0.04 | 0.10 | 0.672 | 0.42 | 0.40 | 0.292 | 0.07 | 0.15 | 0.655 |
|  | NA intensity | 0.35 | 0.56 | 0.528 | -0.14 | 0.08 | 0.088 | -0.13 | 0.34 | 0.702 | 0.27 | 0.11 | 0.020 |
|  | PA variability | -0.08 | 0.41 | 0.850 | -0.10 | 0.07 | 0.144 | 0.21 | 0.41 | 0.605 | 0.09 | 0.11 | 0.415 |
|  | NA variability | 0.17 | 0.37 | 0.652 | -0.10 | 0.07 | 0.158 | 0.14 | 0.40 | 0.724 | 0.10 | 0.10 | 0.301 |
|  | | *M* | *SE* | *p* | *M* | *SE* | *p* | *M* | *SE* | *p* | *M* | *SE* | *p* |
| Social support | PA intensity | 0.81 | 0.36 | 0.023 | 0.02 | 0.04 | 0.697 | -0.14 | 0.15 | 0.350 | 0.01 | 0.06 | 0.903 |
|  | NA intensity | -0.29 | 0.21 | 0.173 | -0.01 | 0.03 | 0.740 | 0.11 | 0.13 | 0.418 | 0.01 | 0.05 | 0.872 |
|  | PA variability | -0.25 | 0.15 | 0.100 | 0.02 | 0.03 | 0.548 | -0.06 | 0.16 | 0.710 | 0.02 | 0.04 | 0.689 |
|  | NA variability | -0.11 | 0.14 | 0.443 | 0.00 | 0.03 | 0.996 | -0.05 | 0.16 | 0.744 | 0.02 | 0.04 | 0.608 |
|  | | *M* | *SE* | *p* | *M* | *SE* | *p* | *M* | *SE* | *p* | *M* | *SE* | *p* |
| Palliative reaction | PA intensity | -0.49 | 0.61 | 0.422 | -0.01 | 0.07 | 0.880 | 0.13 | 0.26 | 0.611 | 0.05 | 0.10 | 0.620 |
|  | NA intensity | 0.39 | 0.36 | 0.276 | 0.01 | 0.06 | 0.817 | -0.01 | 0.22 | 0.971 | 0.02 | 0.08 | 0.749 |
|  | PA variability | 0.01 | 0.23 | 0.951 | 0.01 | 0.04 | 0.912 | 0.11 | 0.25 | 0.659 | 0.02 | 0.06 | 0.776 |
|  | NA variability | 0.19 | 0.24 | 0.421 | 0.01 | 0.05 | 0.829 | 0.17 | 0.26 | 0.507 | -0.06 | 0.06 | 0.344 |

*Note.* PA= positive affect, NA = negative affect. L1= level 1, L2 = level 2, S1= slope 1, S2= slope 2. L1 reflects the general level of affect intensity or variability before the lockdown. S1 reflects the general changes during the whole study period. L2 reflects the immediate change in intensity or variability the week before the lockdown and the first week of the lockdown. S2 reflects the gradual changes during the lockdown weeks, above and beyond the S1. Significant effects (p<.01) are highlighted in bold.

**Table S8.** Model fit of the sensitivity models.

|  | **Adolescents** | | | | **Parents** | | | | |
| --- | --- | --- | --- | --- | --- | --- | --- | --- | --- |
|  | **RMSEA** | **CFI** | **TLI** |  | | **RMSEA** | **CFI** | **TLI** |  |
| PA intensity | 0.134 (.12-.15) | 0.926 | 0.926 | Acceptable | | 0.094 (.08-.11) | 0.964 | 0.964 | Acceptable |
| NA intensity | 0.106 (.09-.12) | 0.940 | 0.940 | Acceptable | | 0.097 (.08-.11) | 0.944 | 0.944 | Acceptable |
| PA variability | 0.056 (.04-.08) | 0.906 | 0.906 | Acceptable | | 0.046 (.02-.07) | 0.950 | 0.950 | Acceptable |
| NA variability | 0.071 (.05-.09) | 0.925 | 0.925 | Acceptable | | 0.076 (.06-.09) | 0.909 | 0.909 | Acceptable |

Note: PA= positive affect, NA = negative affect. RMSEA= root mean square error of approximation, CFI= comparative fit index, TLI= Tucker-Lewis index.

**Table S9.** Estimates from the sensitivity model.

|  |  | **Adolescents** | | | |  |  |  | **Parents** |  |  |  |  |
| --- | --- | --- | --- | --- | --- | --- | --- | --- | --- | --- | --- | --- | --- |
| **Variable** |  | *M* | *SE* | *p* | **Variance** | *SE* | *p* | **Mean** | *SE* | *p* | **Variance** | *SE* | *p* |
| PA intensity | L1 | 76.22 | 1.46 | <.001 | 318.36 | 38.03 | **<.001** | 70.22 | 1.39 | <.001 | 292.68 | 34.34 | **<.001** |
|  | S1 | 0.03 | 0.14 | 0.834 | 1.61 | 0.38 | **<.001** | -0.02 | 0.15 | 0.902 | 2.15 | 0.39 | **<.001** |
|  | L2 | 1.36 | 0.71 | 0.055 | 30.62 | 8.44 | **<.001** | 1.49 | 0.65 | **0.023** | 26.35 | 7.62 | 0.001 |
|  | S2 | -0.26 | 0.23 | 0.266 | 5.47 | 0.98 | **<.001** | -0.38 | 0.21 | 0.072 | 4.35 | 0.80 | **<.001** |
| NA intensity | L1 | 10.47 | 0.93 | <.001 | 120.30 | 15.08 | **<.001** | 9.38 | 0.83 | <.001 | 100.90 | 12.21 | **<.001** |
|  | S1 | -0.30 | 0.12 | **0.012** | 1.15 | 0.26 | **<.001** | -0.31 | 0.12 | **0.011** | 1.67 | 0.27 | **<.001** |
|  | L2 | -0.49 | 0.53 | 0.355 | 11.18 | 4.79 | **0.020** | -0.21 | 0.53 | 0.698 | 19.47 | 5.18 | **<.001** |
|  | S2 | 0.53 | 0.17 | **0.002** | 2.73 | 0.53 | **<.001** | 0.66 | 0.17 | **<.001** | 2.57 | 0.50 | **<.001** |
| PA variability | L1 | 11.15 | 0.74 | <.001 | 67.64 | 10.07 | **<.001** | 10.10 | 0.56 | <.001 | 34.02 | 5.77 | **<.001** |
|  | S1 | -0.51 | 0.13 | **<.001** | 1.05 | 0.36 | **0.003** | -0.68 | 0.12 | **<.001** | 0.73 | 0.27 | **0.006** |
|  | L2 | 0.75 | 0.65 | 0.245 | 11.19 | 8.57 | 0.191 | 1.14 | 0.63 | 0.070 | 16.82 | 7.56 | **0.026** |
|  | S2 | 0.16 | 0.19 | 0.398 | 2.15 | 0.70 | **0.002** | 0.54 | 0.16 | **0.001** | 1.36 | 0.52 | **0.009** |
| NA variability | L1 | 10.68 | 0.69 | <.001 | 49.30 | 8.61 | **<.001** | 8.85 | 0.56 | <.001 | 32.12 | 5.75 | **<.001** |
|  | S1 | -0.53 | 0.13 | **<.001** | 0.86 | 0.33 | **0.010** | -0.58 | 0.11 | **<.001** | 0.47 | 0.23 | **0.039** |
|  | L2 | -0.62 | 0.67 | 0.360 | 6.15 | 8.33 | 0.460 | 0.76 | 0.63 | 0.232 | 12.59 | 7.59 | 0.097 |
|  | S2 | 0.61 | 0.20 | **0.002** | 2.05 | 0.70 | **0.004** | 0.65 | 0.15 | **<.001** | 0.12 | 0.40 | 0.763 |

Note: PA= positive affect, NA = negative affect. L1= level 1, L2 = level 2, S1= slope 1, S2= slope 2.

**Table S10.** Correlations (95% CI) between the estimated intercepts and slopes of the adolescent and their parent.

|  | **L1** |  | **S1** |  | **L2** |  | **S2** |  |
| --- | --- | --- | --- | --- | --- | --- | --- | --- |
|  | *r*  *(95%CI)* | *p* | *r*  *(95%CI)* | *p* | *r*  *(95%CI)* | *p* | *r*  *(95%CI)* | *p* |
| PA intensity | **.27**  (.11-.40) | **.001** | 0.18  (.02 - .32) | .027 | 0.05  (-.11- .20) | .533 | 0.14  (-.02- .29) | .078 |
| NA intensity | -.002  (-.15- .15) | .981 | -0.06  (-.21 - .09) | .443 | 0.01  (-.14 -.17) | .888 | -0.05  (-.20 - .11) | .565 |
| PA variability | .05  (-.10 - .20) | .526 | 0.05  (-.11 - .20) | .569 | 0.10  (-.06 - .25) | .220 | 0.03  (-.13 - .18) | .722 |
| NA variability | -.03  (-.18 - .13) | .728 | 0.15  (-.01 - .29) | .068 | **0.20**  (.05 - .35) | **.011** | 0.03  (-.12 - .19) | .670 |

Note: PA= positive affect, NA = negative affect. L1= level 1, L2 = level 2, S1= slope 1, S2= slope 2.

**Covariance matrices**

**Table S11.** Covariance matrix of positive affect intensity for adolescents.

|  | LD -6 | LD -5 | LD -4 | LD -3 | LD -2 | LD -1 | LD 0 | LD +1 | LD +2 | LD +3 | LD +4 | LD +5 | LD +6 | LD +7 |
| --- | --- | --- | --- | --- | --- | --- | --- | --- | --- | --- | --- | --- | --- | --- |
| LD -6 | 1.000 |  |  |  |  |  |  |  |  |  |  |  |  |  |
| LD -5 | 1.000 | 1.000 |  |  |  |  |  |  |  |  |  |  |  |  |
| LD -4 | 1.000 | 1.000 | 1.000 |  |  |  |  |  |  |  |  |  |  |  |
| LD -3 | 0.994 | 0.994 | 0.994 | 0.994 |  |  |  |  |  |  |  |  |  |  |
| LD -2 | 1.000 | 1.000 | 1.000 | 0.994 | 1.000 |  |  |  |  |  |  |  |  |  |
| LD -1 | 0.994 | 0.994 | 0.994 | 0.987 | 0.994 | 0.994 |  |  |  |  |  |  |  |  |
| LD 0 | 0.987 | 0.987 | 0.987 | 0.981 | 0.987 | 0.981 | 0.987 |  |  |  |  |  |  |  |
| LD +1 | 0.981 | 0.981 | 0.981 | 0.975 | 0.981 | 0.981 | 0.969 | 0.981 |  |  |  |  |  |  |
| LD +2 | 0.987 | 0.987 | 0.987 | 0.981 | 0.987 | 0.987 | 0.975 | 0.975 | 0.987 |  |  |  |  |  |
| LD +3 | 0.969 | 0.969 | 0.969 | 0.962 | 0.969 | 0.969 | 0.969 | 0.956 | 0.969 | 0.969 |  |  |  |  |
| LD +4 | 0.969 | 0.969 | 0.969 | 0.962 | 0.969 | 0.969 | 0.969 | 0.962 | 0.962 | 0.962 | 0.969 |  |  |  |
| LD +5 | 0.975 | 0.975 | 0.975 | 0.969 | 0.975 | 0.975 | 0.975 | 0.962 | 0.969 | 0.969 | 0.969 | 0.975 |  |  |
| LD +6 | 0.975 | 0.975 | 0.975 | 0.969 | 0.975 | 0.975 | 0.969 | 0.969 | 0.969 | 0.962 | 0.962 | 0.969 | 0.975 |  |
| LD +7 | 0.956 | 0.956 | 0.956 | 0.950 | 0.956 | 0.956 | 0.956 | 0.950 | 0.956 | 0.956 | 0.950 | 0.956 | 0.956 | 0.956 |

*Note*: LD = lockdown. LD-6 is seven weeks before the start of the lockdown, LD-5 is six weeks before the start of the lockdown, etc, LD0 is the week before the lockdown, LD+1 is the first week of the lockdown, LD+2 is the second week of the lockdown, etc.

**Table S12.** Covariance matrix of negative affect intensity for adolescents.

|  | LD -6 | LD -5 | LD -4 | LD -3 | LD -2 | LD -1 | LD 0 | LD +1 | LD +2 | LD +3 | LD +4 | LD +5 | LD +6 | LD +7 |
| --- | --- | --- | --- | --- | --- | --- | --- | --- | --- | --- | --- | --- | --- | --- |
| LD -6 | 1.000 |  |  |  |  |  |  |  |  |  |  |  |  |  |
| LD -5 | 1.000 | 1.000 |  |  |  |  |  |  |  |  |  |  |  |  |
| LD -4 | 1.000 | 1.000 | 1.000 |  |  |  |  |  |  |  |  |  |  |  |
| LD -3 | 0.994 | 0.994 | 0.994 | 0.994 |  |  |  |  |  |  |  |  |  |  |
| LD -2 | 1.000 | 1.000 | 1.000 | 0.994 | 1.000 |  |  |  |  |  |  |  |  |  |
| LD -1 | 0.994 | 0.994 | 0.994 | 0.987 | 0.994 | 0.994 |  |  |  |  |  |  |  |  |
| LD 0 | 0.987 | 0.987 | 0.987 | 0.981 | 0.987 | 0.981 | 0.987 |  |  |  |  |  |  |  |
| LD +1 | 0.981 | 0.981 | 0.981 | 0.975 | 0.981 | 0.981 | 0.969 | 0.981 |  |  |  |  |  |  |
| LD +2 | 0.987 | 0.987 | 0.987 | 0.981 | 0.987 | 0.987 | 0.975 | 0.975 | 0.987 |  |  |  |  |  |
| LD +3 | 0.969 | 0.969 | 0.969 | 0.962 | 0.969 | 0.969 | 0.969 | 0.956 | 0.969 | 0.969 |  |  |  |  |
| LD +4 | 0.969 | 0.969 | 0.969 | 0.962 | 0.969 | 0.969 | 0.969 | 0.962 | 0.962 | 0.962 | 0.969 |  |  |  |
| LD +5 | 0.975 | 0.975 | 0.975 | 0.969 | 0.975 | 0.975 | 0.975 | 0.962 | 0.969 | 0.969 | 0.969 | 0.975 |  |  |
| LD +6 | 0.975 | 0.975 | 0.975 | 0.969 | 0.975 | 0.975 | 0.969 | 0.969 | 0.969 | 0.962 | 0.962 | 0.969 | 0.975 |  |
| LD +7 | 0.956 | 0.956 | 0.956 | 0.95 | 0.956 | 0.956 | 0.956 | 0.95 | 0.956 | 0.956 | 0.95 | 0.956 | 0.956 | 0.956 |

*Note*: LD = lockdown. LD-6 is seven weeks before the start of the lockdown, LD-5 is six weeks before the start of the lockdown, etc, LD0 is the week before the lockdown, LD+1 is the first week of the lockdown, LD+2 is the second week of the lockdown, etc.

**Table S13**. Covariance matrix of positive affect variability for adolescents.

|  | LD -6 | LD -5 | LD -4 | LD -3 | LD -2 | LD -1 | LD 0 | LD +1 | LD +2 | LD +3 | LD +4 | LD +5 | LD +6 | LD +7 |
| --- | --- | --- | --- | --- | --- | --- | --- | --- | --- | --- | --- | --- | --- | --- |
| LD -6 | 1.000 |  |  |  |  |  |  |  |  |  |  |  |  |  |
| LD -5 | 1.000 | 1.000 |  |  |  |  |  |  |  |  |  |  |  |  |
| LD -4 | 1.000 | 1.000 | 1.000 |  |  |  |  |  |  |  |  |  |  |  |
| LD -3 | 0.994 | 0.994 | 0.994 | 0.994 |  |  |  |  |  |  |  |  |  |  |
| LD -2 | 1.000 | 1.000 | 1.000 | 0.994 | 1.000 |  |  |  |  |  |  |  |  |  |
| LD -1 | 0.987 | 0.987 | 0.987 | 0.981 | 0.987 | 0.987 |  |  |  |  |  |  |  |  |
| LD 0 | 0.987 | 0.987 | 0.987 | 0.987 | 0.987 | 0.975 | 0.987 |  |  |  |  |  |  |  |
| LD +1 | 0.994 | 0.994 | 0.994 | 0.987 | 0.994 | 0.981 | 0.981 | 0.994 |  |  |  |  |  |  |
| LD +2 | 0.975 | 0.975 | 0.975 | 0.969 | 0.975 | 0.969 | 0.969 | 0.969 | 0.975 |  |  |  |  |  |
| LD +3 | 1.000 | 1.000 | 1.000 | 0.994 | 1.000 | 0.987 | 0.987 | 0.994 | 0.975 | 1.000 |  |  |  |  |
| LD +4 | 1.000 | 1.000 | 1.000 | 0.994 | 1.000 | 0.987 | 0.987 | 0.994 | 0.975 | 1.000 | 1.000 |  |  |  |
| LD +5 | 0.994 | 0.994 | 0.994 | 0.987 | 0.994 | 0.981 | 0.981 | 0.987 | 0.969 | 0.994 | 0.994 | 0.994 |  |  |
| LD +6 | 0.969 | 0.969 | 0.969 | 0.962 | 0.969 | 0.956 | 0.956 | 0.962 | 0.95 | 0.969 | 0.969 | 0.969 | 0.969 |  |
| LD +7 | 0.987 | 0.987 | 0.987 | 0.981 | 0.987 | 0.975 | 0.975 | 0.981 | 0.962 | 0.987 | 0.987 | 0.987 | 0.962 | 0.987 |

*Note*: LD = lockdown. LD-6 is seven weeks before the start of the lockdown, LD-5 is six weeks before the start of the lockdown, etc, LD0 is the week before the lockdown, LD+1 is the first week of the lockdown, LD+2 is the second week of the lockdown, etc.

**Table S14.** Covariance matrix of negative affect variability for adolescents.

|  | LD -6 | LD -5 | LD -4 | LD -3 | LD -2 | LD -1 | LD 0 | LD +1 | LD +2 | LD +3 | LD +4 | LD +5 | LD +6 | LD +7 |
| --- | --- | --- | --- | --- | --- | --- | --- | --- | --- | --- | --- | --- | --- | --- |
| LD -6 | 1.000 |  |  |  |  |  |  |  |  |  |  |  |  |  |
| LD -5 | 1.000 | 1.000 |  |  |  |  |  |  |  |  |  |  |  |  |
| LD -4 | 1.000 | 1.000 | 1.000 |  |  |  |  |  |  |  |  |  |  |  |
| LD -3 | 0.994 | 0.994 | 0.994 | 0.994 |  |  |  |  |  |  |  |  |  |  |
| LD -2 | 1.000 | 1.000 | 1.000 | 0.994 | 1.000 |  |  |  |  |  |  |  |  |  |
| LD -1 | 0.987 | 0.987 | 0.987 | 0.981 | 0.987 | 0.987 |  |  |  |  |  |  |  |  |
| LD 0 | 0.987 | 0.987 | 0.987 | 0.987 | 0.987 | 0.975 | 0.987 |  |  |  |  |  |  |  |
| LD +1 | 0.994 | 0.994 | 0.994 | 0.987 | 0.994 | 0.981 | 0.981 | 0.994 |  |  |  |  |  |  |
| LD +2 | 0.975 | 0.975 | 0.975 | 0.969 | 0.975 | 0.969 | 0.969 | 0.969 | 0.975 |  |  |  |  |  |
| LD +3 | 1.000 | 1.000 | 1.000 | 0.994 | 1.000 | 0.987 | 0.987 | 0.994 | 0.975 | 1.000 |  |  |  |  |
| LD +4 | 1.000 | 1.000 | 1.000 | 0.994 | 1.000 | 0.987 | 0.987 | 0.994 | 0.975 | 1.000 | 1.000 |  |  |  |
| LD +5 | 0.994 | 0.994 | 0.994 | 0.987 | 0.994 | 0.981 | 0.981 | 0.987 | 0.969 | 0.994 | 0.994 | 0.994 |  |  |
| LD +6 | 0.969 | 0.969 | 0.969 | 0.962 | 0.969 | 0.956 | 0.956 | 0.962 | 0.950 | 0.969 | 0.969 | 0.969 | 0.969 |  |
| LD +7 | 0.987 | 0.987 | 0.987 | 0.981 | 0.987 | 0.975 | 0.975 | 0.981 | 0.962 | 0.987 | 0.987 | 0.987 | 0.962 | 0.987 |

*Note*: LD = lockdown. LD-6 is seven weeks before the start of the lockdown, LD-5 is six weeks before the start of the lockdown, etc, LD0 is the week before the lockdown, LD+1 is the first week of the lockdown, LD+2 is the second week of the lockdown, etc.

**Table S15.** Covariance matrix of positive affect intensity for parents.

|  | LD -6 | LD -5 | LD -4 | LD -3 | LD -2 | LD -1 | LD 0 | LD +1 | LD +2 | LD +3 | LD +4 | LD +5 | LD +6 | LD +7 |
| --- | --- | --- | --- | --- | --- | --- | --- | --- | --- | --- | --- | --- | --- | --- |
| LD -6 | 1.000 |  |  |  |  |  |  |  |  |  |  |  |  |  |
| LD -5 | 1.000 | 1.000 |  |  |  |  |  |  |  |  |  |  |  |  |
| LD -4 | 1.000 | 1.000 | 1.000 |  |  |  |  |  |  |  |  |  |  |  |
| LD -3 | 1.000 | 1.000 | 1.000 | 1.000 |  |  |  |  |  |  |  |  |  |  |
| LD -2 | 1.000 | 1.000 | 1.000 | 1.000 | 1.000 |  |  |  |  |  |  |  |  |  |
| LD -1 | 1.000 | 1.000 | 1.000 | 1.000 | 1.000 | 1.000 |  |  |  |  |  |  |  |  |
| LD 0 | 0.994 | 0.994 | 0.994 | 0.994 | 0.994 | 0.994 | 0.994 |  |  |  |  |  |  |  |
| LD +1 | 0.994 | 0.994 | 0.994 | 0.994 | 0.994 | 0.994 | 0.987 | 0.994 |  |  |  |  |  |  |
| LD +2 | 0.981 | 0.981 | 0.981 | 0.981 | 0.981 | 0.981 | 0.981 | 0.981 | 0.981 |  |  |  |  |  |
| LD +3 | 0.987 | 0.987 | 0.987 | 0.987 | 0.987 | 0.987 | 0.987 | 0.987 | 0.981 | 0.987 |  |  |  |  |
| LD +4 | 0.981 | 0.981 | 0.981 | 0.981 | 0.981 | 0.981 | 0.981 | 0.981 | 0.975 | 0.981 | 0.981 |  |  |  |
| LD +5 | 0.975 | 0.975 | 0.975 | 0.975 | 0.975 | 0.975 | 0.975 | 0.975 | 0.975 | 0.975 | 0.975 | 0.975 |  |  |
| LD +6 | 0.981 | 0.981 | 0.981 | 0.981 | 0.981 | 0.981 | 0.981 | 0.975 | 0.969 | 0.975 | 0.975 | 0.969 | 0.981 |  |
| LD +7 | 0.981 | 0.981 | 0.981 | 0.981 | 0.981 | 0.981 | 0.981 | 0.975 | 0.969 | 0.975 | 0.975 | 0.969 | 0.975 | 0.981 |

*Note*: LD = lockdown. LD-6 is seven weeks before the start of the lockdown, LD-5 is six weeks before the start of the lockdown, etc, LD0 is the week before the lockdown, LD+1 is the first week of the lockdown, LD+2 is the second week of the lockdown, etc.

**Table S16.** Covariance matrix of negative affect intensity for parents.

|  | LD -6 | LD -5 | LD -4 | LD -3 | LD -2 | LD -1 | LD 0 | LD +1 | LD +2 | LD +3 | LD +4 | LD +5 | LD +6 | LD +7 |
| --- | --- | --- | --- | --- | --- | --- | --- | --- | --- | --- | --- | --- | --- | --- |
| LD -6 | 1.000 |  |  |  |  |  |  |  |  |  |  |  |  |  |
| LD -5 | 1.000 | 1.000 |  |  |  |  |  |  |  |  |  |  |  |  |
| LD -4 | 1.000 | 1.000 | 1.000 |  |  |  |  |  |  |  |  |  |  |  |
| LD -3 | 1.000 | 1.000 | 1.000 | 1.000 |  |  |  |  |  |  |  |  |  |  |
| LD -2 | 1.000 | 1.000 | 1.000 | 1.000 | 1.000 |  |  |  |  |  |  |  |  |  |
| LD -1 | 1.000 | 1.000 | 1.000 | 1.000 | 1.000 | 1.000 |  |  |  |  |  |  |  |  |
| LD 0 | 0.994 | 0.994 | 0.994 | 0.994 | 0.994 | 0.994 | 0.994 |  |  |  |  |  |  |  |
| LD +1 | 0.994 | 0.994 | 0.994 | 0.994 | 0.994 | 0.994 | 0.987 | 0.994 |  |  |  |  |  |  |
| LD +2 | 0.981 | 0.981 | 0.981 | 0.981 | 0.981 | 0.981 | 0.981 | 0.981 | 0.981 |  |  |  |  |  |
| LD +3 | 0.987 | 0.987 | 0.987 | 0.987 | 0.987 | 0.987 | 0.987 | 0.987 | 0.981 | 0.987 |  |  |  |  |
| LD +4 | 0.981 | 0.981 | 0.981 | 0.981 | 0.981 | 0.981 | 0.981 | 0.981 | 0.975 | 0.981 | 0.981 |  |  |  |
| LD +5 | 0.975 | 0.975 | 0.975 | 0.975 | 0.975 | 0.975 | 0.975 | 0.975 | 0.975 | 0.975 | 0.975 | 0.975 |  |  |
| LD +6 | 0.981 | 0.981 | 0.981 | 0.981 | 0.981 | 0.981 | 0.981 | 0.975 | 0.969 | 0.975 | 0.975 | 0.969 | 0.981 |  |
| LD +7 | 0.981 | 0.981 | 0.981 | 0.981 | 0.981 | 0.981 | 0.981 | 0.975 | 0.969 | 0.975 | 0.975 | 0.969 | 0.975 | 0.981 |

*Note*: LD = lockdown. LD-6 is seven weeks before the start of the lockdown, LD-5 is six weeks before the start of the lockdown, etc, LD0 is the week before the lockdown, LD+1 is the first week of the lockdown, LD+2 is the second week of the lockdown, etc.

**Table S17.** Covariance matrix of positive affect variability for parents.

|  | LD -6 | LD -5 | LD -4 | LD -3 | LD -2 | LD -1 | LD 0 | LD +1 | LD +2 | LD +3 | LD +4 | LD +5 | LD +6 | LD +7 |
| --- | --- | --- | --- | --- | --- | --- | --- | --- | --- | --- | --- | --- | --- | --- |
| LD -6 | 1.000 |  |  |  |  |  |  |  |  |  |  |  |  |  |
| LD -5 | 1.000 | 1.000 |  |  |  |  |  |  |  |  |  |  |  |  |
| LD -4 | 1.000 | 1.000 | 1.000 |  |  |  |  |  |  |  |  |  |  |  |
| LD -3 | 1.000 | 1.000 | 1.000 | 1.000 |  |  |  |  |  |  |  |  |  |  |
| LD -2 | 0.994 | 0.994 | 0.994 | 0.994 | 0.994 |  |  |  |  |  |  |  |  |  |
| LD -1 | 1.000 | 1.000 | 1.000 | 1.000 | 0.994 | 1.000 |  |  |  |  |  |  |  |  |
| LD 0 | 1.000 | 1.000 | 1.000 | 1.000 | 0.994 | 1.000 | 1.000 |  |  |  |  |  |  |  |
| LD +1 | 1.000 | 1.000 | 1.000 | 1.000 | 0.994 | 1.000 | 1.000 | 1.000 |  |  |  |  |  |  |
| LD +2 | 0.994 | 0.994 | 0.994 | 0.994 | 0.987 | 0.994 | 0.994 | 0.994 | 0.994 |  |  |  |  |  |
| LD +3 | 0.994 | 0.994 | 0.994 | 0.994 | 0.987 | 0.994 | 0.994 | 0.994 | 0.987 | 0.994 |  |  |  |  |
| LD +4 | 1.000 | 1.000 | 1.000 | 1.000 | 0.994 | 1.000 | 1.000 | 1.000 | 0.994 | 0.994 | 1.000 |  |  |  |
| LD +5 | 1.000 | 1.000 | 1.000 | 1.000 | 0.994 | 1.000 | 1.000 | 1.000 | 0.994 | 0.994 | 1.000 | 1.000 |  |  |
| LD +6 | 0.994 | 0.994 | 0.994 | 0.994 | 0.987 | 0.994 | 0.994 | 0.994 | 0.987 | 0.987 | 0.994 | 0.994 | 0.994 |  |
| LD +7 | 0.987 | 0.987 | 0.987 | 0.987 | 0.981 | 0.987 | 0.987 | 0.987 | 0.981 | 0.981 | 0.987 | 0.987 | 0.981 | 0.987 |

*Note*: LD = lockdown. LD-6 is seven weeks before the start of the lockdown, LD-5 is six weeks before the start of the lockdown, etc, LD0 is the week before the lockdown, LD+1 is the first week of the lockdown, LD+2 is the second week of the lockdown, etc.

**Table S18.** Covariance matrix of negative affect variability for parents.

|  | LD -6 | LD -5 | LD -4 | LD -3 | LD -2 | LD -1 | LD 0 | LD +1 | LD +2 | LD +3 | LD +4 | LD +5 | LD +6 | LD +7 |
| --- | --- | --- | --- | --- | --- | --- | --- | --- | --- | --- | --- | --- | --- | --- |
| LD -6 | 1.000 |  |  |  |  |  |  |  |  |  |  |  |  |  |
| LD -5 | 1.000 | 1.000 |  |  |  |  |  |  |  |  |  |  |  |  |
| LD -4 | 1.000 | 1.000 | 1.000 |  |  |  |  |  |  |  |  |  |  |  |
| LD -3 | 1.000 | 1.000 | 1.000 | 1.000 |  |  |  |  |  |  |  |  |  |  |
| LD -2 | 0.994 | 0.994 | 0.994 | 0.994 | 0.994 |  |  |  |  |  |  |  |  |  |
| LD -1 | 1.000 | 1.000 | 1.000 | 1.000 | 0.994 | 1.000 |  |  |  |  |  |  |  |  |
| LD 0 | 1.000 | 1.000 | 1.000 | 1.000 | 0.994 | 1.000 | 1.000 |  |  |  |  |  |  |  |
| LD +1 | 1.000 | 1.000 | 1.000 | 1.000 | 0.994 | 1.000 | 1.000 | 1.000 |  |  |  |  |  |  |
| LD +2 | 0.994 | 0.994 | 0.994 | 0.994 | 0.987 | 0.994 | 0.994 | 0.994 | 0.994 |  |  |  |  |  |
| LD +3 | 0.994 | 0.994 | 0.994 | 0.994 | 0.987 | 0.994 | 0.994 | 0.994 | 0.987 | 0.994 |  |  |  |  |
| LD +4 | 1.000 | 1.000 | 1.000 | 1.000 | 0.994 | 1.000 | 1.000 | 1.000 | 0.994 | 0.994 | 1.000 |  |  |  |
| LD +5 | 1.000 | 1.000 | 1.000 | 1.000 | 0.994 | 1.000 | 1.000 | 1.000 | 0.994 | 0.994 | 1.000 | 1.000 |  |  |
| LD +6 | 0.994 | 0.994 | 0.994 | 0.994 | 0.987 | 0.994 | 0.994 | 0.994 | 0.987 | 0.987 | 0.994 | 0.994 | 0.994 |  |
| LD +7 | 0.987 | 0.987 | 0.987 | 0.987 | 0.981 | 0.987 | 0.987 | 0.987 | 0.981 | 0.981 | 0.987 | 0.987 | 0.981 | 0.987 |

*Note*: LD = lockdown. LD-6 is seven weeks before the start of the lockdown, LD-5 is six weeks before the start of the lockdown, etc, LD0 is the week before the lockdown, LD+1 is the first week of the lockdown, LD+2 is the second week of the lockdown, etc.

**Mplus Scripts**

1. **Main models (H1, H3)**

TITLE: Piecewise growth model two phases

DATA: FILE IS C:\Users\Admin\surfdrive\p_rmssdNA_data_y1-14.txt;

VARIABLE: NAMES ARE id y1-y14;

IDVARIABLE = id;

MISSING = ALL(999); !missing values

USEVAR = y1-y14; ,

MODEL:L S|y1@-6 y2@-5 y3@-4 y4@-3 y5@-2 y6@-1 y7@0 y8@1 y9@2 y10@3 y11@4 y12@5 y13@6 y14@7;

L2 S2 | y8@0 y9@1 y10@2 y11@3 y12@4 y13@5 y14@6;

OUTPUT: TECH1 TECH4;

SAVEDATA:

FILE = parameters_p_rmssdNA.dat;

SAVE = fscores;

FORMAT = free;

1. **Constrain L2 or S2 in the parent models to the value of adolescents (H2)**

TITLE: Piecewise growth model two phases

DATA: FILE IS C:\Users\Admin\surfdrive\p_meanPA_data_y1-14.txt;

VARIABLE: NAMES ARE id y1-y14;

IDVARIABLE = id;

MISSING = ALL(999); !missing values

USEVAR = y1-y14;

MODEL:L S|y1@-6 y2@-5 y3@-4 y4@-3 y5@-2 y6@-1 y7@0 y8@1 y9@2 y10@3 y11@4 y12@5 y13@6 y14@7;

L2 S2 | y8@0 y9@1 y10@2 y11@3 y12@4 y13@5 y14@6;

[S2@-0.20];

OUTPUT: TECH1 TECH4;

SAVEDATA:

FILE = parameters_p_meanPA_S2constrain.dat;

SAVE = fscores;

FORMAT = free;

1. **Covariate models (H4)**

TITLE: Piecewise growth model, depr as covariate

DATA: FILE IS C:\Users\Admin\surfdrive\a_rmssdNA_data_depr.txt;

VARIABLE: NAMES ARE id y1-y14 depr;

IDVARIABLE = id;

MISSING = ALL(999); !missing values

USEVAR = y1-y14 depr; ,

MODEL:L S|y1@-6 y2@-5 y3@-4 y4@-3 y5@-2 y6@-1 y7@0 y8@1 y9@2 y10@3 y11@4 y12@5 y13@6 y14@7;

L2 S2 | y8@0 y9@1 y10@2 y11@3 y12@4 y13@5 y14@6;

!confirmatory test of HX

L2 ON depr;

!exploratory tests

L S S2 ON depr;

OUTPUT: TECH1 TECH4;

SAVEDATA:

FILE = parameters_a_rmssdNA_depr.dat;

SAVE = fscores;

FORMAT = free;
